# Supplementary material for: Identification of a diarylpentanoid-producing polyketide synthase revealing an unusual biosynthetic pathway of 2-(2-phenylethyl)chromones in agarwood
Source: Nat Commun. 2022 Jan 17;13:348. doi: 10.1038/s41467-022-27971-z (PMC8764113; doi:10.1038/s41467-022-27971-z)
Supplement: Supplementary file 1 — Supplementary Information [file 41467_2022_27971_MOESM1_ESM.pdf]

Identification of a Diarylpentanoid-Producing Polyketide Synthase Revealing an Unusual Biosynthetic Pathway of 2-(2-Phenylethyl)chromones in Agarwood

Supplementary Information

Supplementary Methods

Supplementary Method 1

General method for synthesis of Acyl-CoAs. Organic acid (5 mM) and *N*-hydroxysuccinimide (NHS, 5 mM) were dissolved in anhydrous tetrahydrofuran (3 mL) and a solution of dicyclohexylcarbodiimide (DCC, 5-10 mM, dehydrating agent) was added dropwise, and the mixture was stirred overnight at room temperature. Then the turbid mixture was filtered and the resulted filtrate was concentrated in vacuo and repeatedly crystallized in ethanol to obtain the intermediate *N*-hydroxysuccinimide esters. Next, coenzyme A (50 mg, 60 μM) dissolved in ice water was added $NaHCO_3$ (25 mg, 0.3 mmol), and a solution of the *N*-hydroxysuccinimide ester (0.12 mmol) in acetone was then added dropwise to the aqueous solution. Finally, the mixture was stirred for thioester exchange at 0 °C for 5 h before being filtered and concentrated in vacuo. The crude product was purified by C18 cartridges to give the corresponding acyl-CoA.

Supplementary Method 2

Synthesis of benzoyl-*β*-diketide acid. To a solution of Meldrum's acid (0.72 g, 0.005 mol) in dry $CHCl_3$ (10 mL) was slowly added dry pyridine (0.9 mL, 0.011 mol) at 0 °C, and the mixture was added dropwise a solution of benzoyl chloride (0.011 mol). After stirring for 1 h at -20 °C and another 1 h at room temperature under nitrogen, the reaction mixture was quenched by slow addition of an aqueous solution of HCl (1 N, 120 mL), and the organic layer was then washed by an aqueous solution of HCl (1 N, 50 mL) before being dried over anhydrous sodium sulfate, filtered and concentrated in vacuo to obtain an oily intermediate.

The acylated intermediate was unstable and was directly used in the next step. Specifically, the oily intermediate (0.005 mol) and sodium methoxide (0.0055 mol) were dissolved in a methanol/H_2O mixture (10/1, v/v, 55 mL), and the mixture was stirred overnight at room temperature. Next day, the residue after vacuum drying was acidified with an aqueous solution of HCl (1 N, 30 mL), and then extracted with dichloromethane (3 × 50 mL), dried over anhydrous sodium sulfate and evaporated. The concentrated residue was dissolved inmethanol and filtered with a 0.22 μm filter. Then the crude product was purified by semi-preparative HPLC to provide benzoyl-*β*-diketide. (Note: *β*-keto acid is unstable at room temperature and tends to decarboxylate).

Synthesis of 4-hydroxyphenylpropionyl-*β*-diketide acid. 4-hydroxyphenylpropionyl-*β*-diketide acid was synthesized by the same strategy of benzoyl-*β*-diketide acid using 3-(4-(benzyloxy)phenyl)propionyl chloride. For the removal of the protect group, a stirred solution of 3-(4-(benzyloxy)phenyl)propionyl-*β*-diketide acid (0.30 g, 1.11 mmol) in EtOAc (3 mL) was added Pd/C 10% (30 mg), and the reaction was stirred for 16 h at room temperature under hydrogen. Then the mixture was filtered and the filtrate was concentrated in vacuo to provide 4-hydroxyphenylpropionyl-*β*-diketide acid.

Supplementary Figures

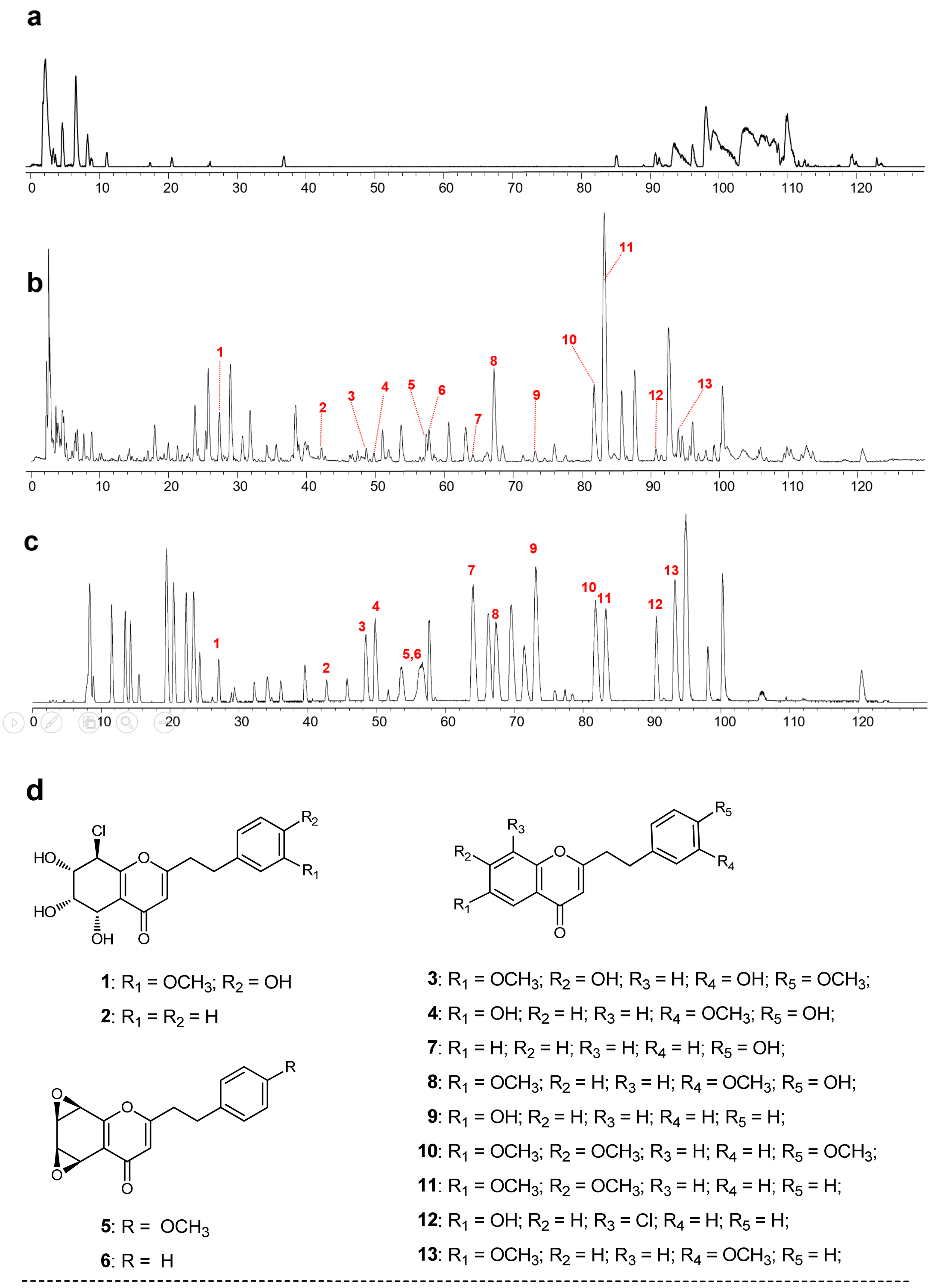

Figure 1. PECs produced by *A. sinensis* calli. **a** MS chromatogram (BPCs) of robustly growing calli. **b** MS chromatogram (BPCs) of 150 mM NaCl-treated calli. **c** MS chromatogram (BPCs) of PECs standards isolated from agarwood (note: only PECs detected from *A. sinensis* calli are presented with numbers). **d** PECs (**1**–**13**) identified from 150 mM NaCl-treated calli by comparisons of their column retention times and MS data with those of standard PECs.

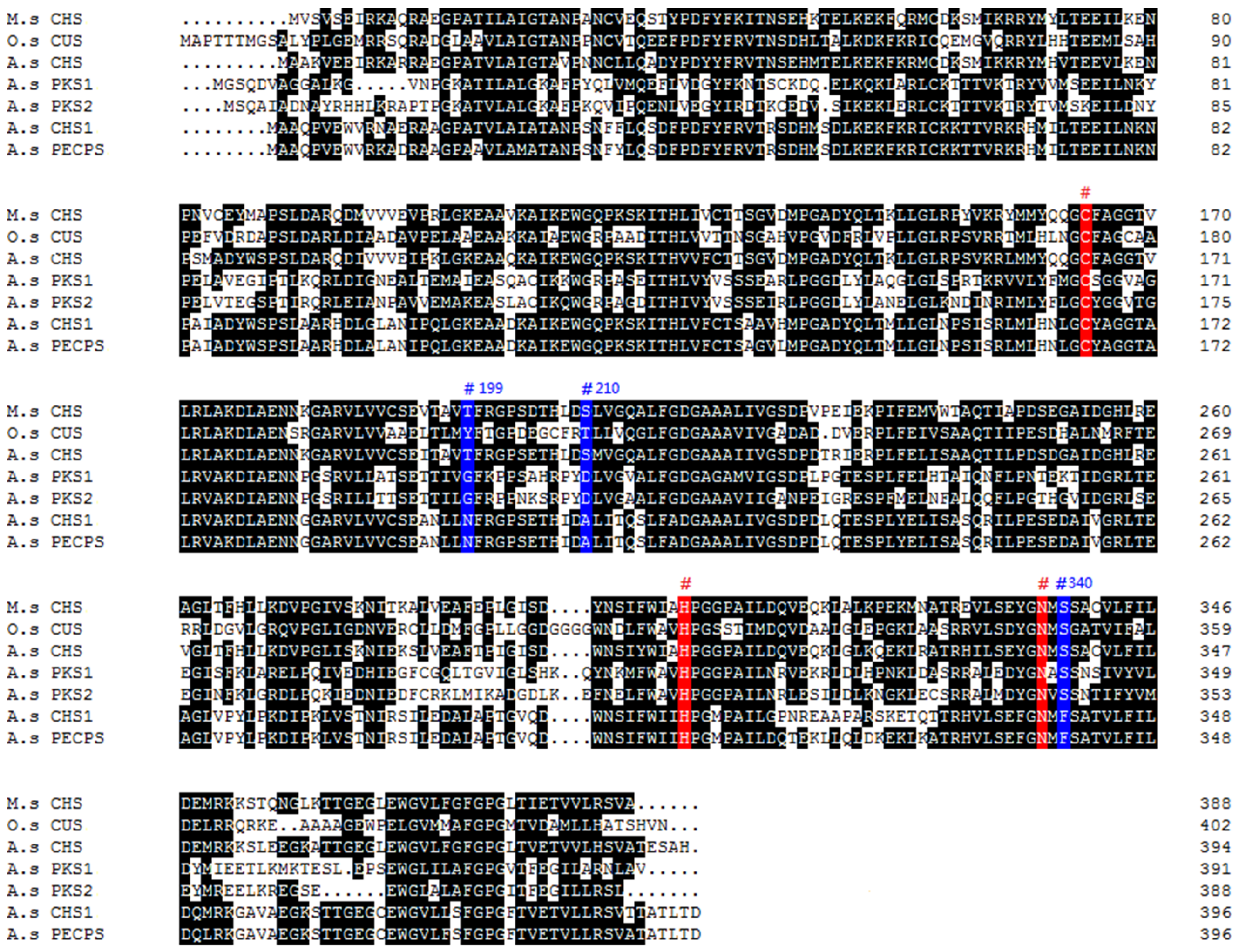

Figure 2. Comparison of the amino acid sequences of PECPS and other CHS superfamily type III PKSs. The alignment was made with ClustalW and the DNAMAN program. M.s CHS, *Medicago sativa* CHS2; O.s CUS, *Oryza sativa* CUS; A.s CHS, *Aquilaria sinensis* CHS; A.s PKS1, *Aquilaria sinensis* PKS1; A.s PKS2, *Aquilaria sinensis* PKS2; A.s CHS1, *Aquilaria sinensis* CHS1; A.s PECPS, *Aquilaria sinensis* PECPS. The catalytic residues conserved in the CHS superfamily enzymes (Cys164, His303, and Asn336, *M. sativa* CHS2 numbering) are highlighted in red. The critical active-site residues 199, 210, and 340 are highlighted in blue. PECPS shares 60%, 47%, 65%, 39%, 37%, and 93% amino acid identity with M.s CHS, O.s CUS, A.s CHS, A.s PKS1, A.s PKS2, and A.s CHS1, respectively, and maintains the Cys-His-Asn catalytic triad conserved in all known type III PKSs.

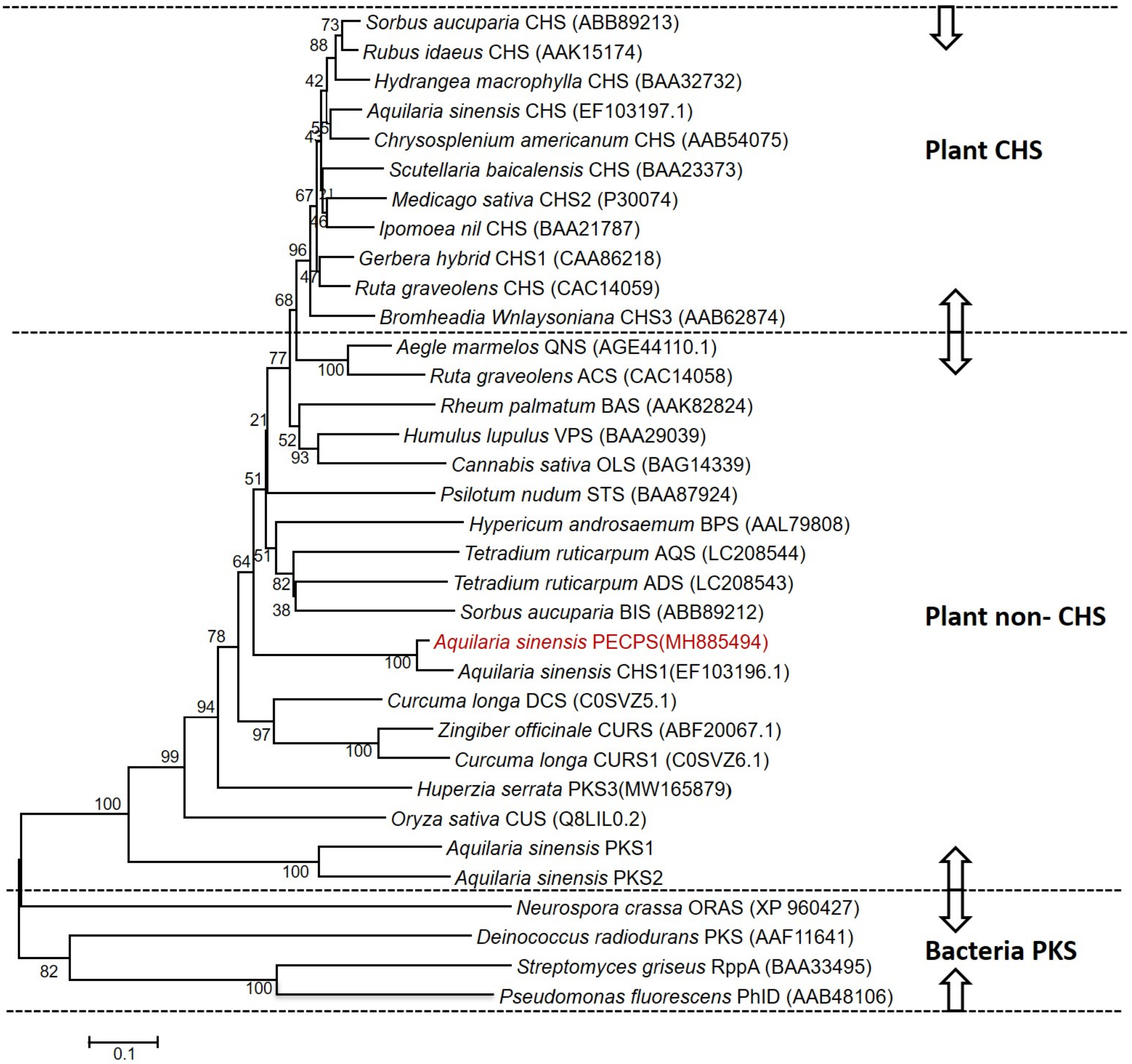

Figure 3. Phylogenetic tree analysis of type III PKSs. Alignment of sequence was generated with ClustalW, and the phylogenetic tree was constructed with MEGA6.0 software using neighbor-joining algorithm. Numbers at the nodes indicates percent bootstrap values. The bar of bottom shows 0.1 amino acid substitution. The reliability of the tree was measured by bootstrap analysis with 1000 replicates. PECPS is highlighted with red. CHS, chalcone synthase; QNS, quinolone synthase; ACS, acridone synthase; BAS, benzalacetone synthase; VPS, valerophenone synthase; OLS, olivetol synthase; STS, stilbene synthase; BPS, benzophenone synthase; AQS, alkylquinolone synthase; ADS: alkyldiketide-CoA synthase; BIS, biphenyl synthase; DCS, diketide-CoA synthase; CURS, curcumin synthase; CUS, curcuminoid synthase; PKS, polyketide synthase; GenBankTM registration numbers are shown in parentheses. **Note:** Although *Aquilaria sinensis* CHS1 (EF103196.1) here was named as CHS, its catalytic function has never been identified, and AsCHS1 was named by the submitter from another group when they submitted this gene to GeneBank.

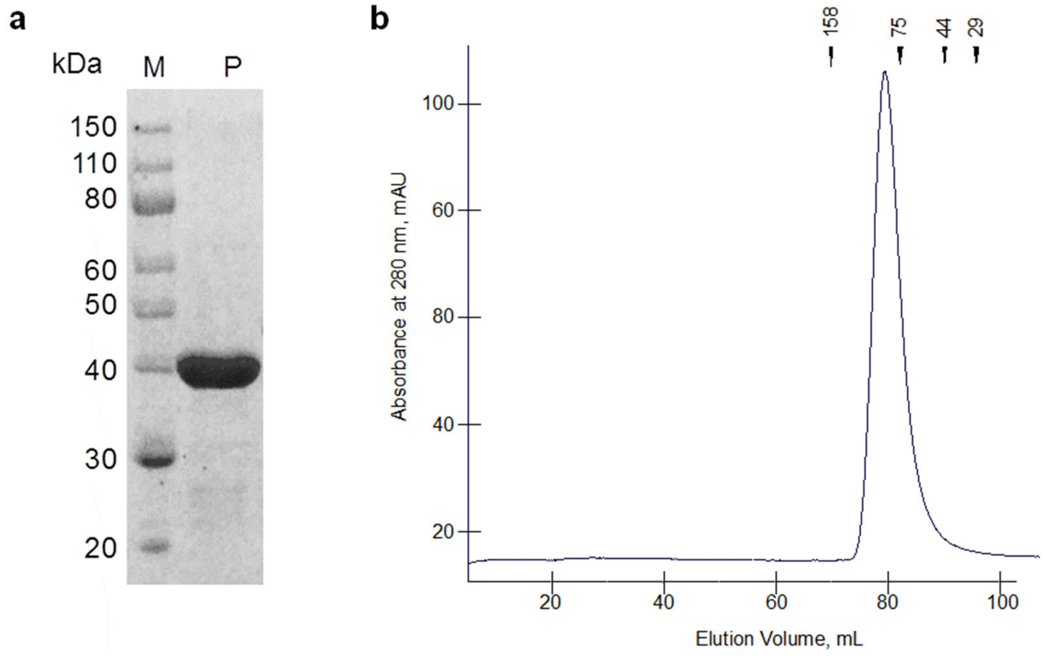

Figure 4. Analyses of the molecular weight of the recombinant PECPS. **a** SDS-PAGE (Lane M, molecular weight markers; lane P, peak fraction). **b** Size-exclusion chromatography (The peak, the recombinant PECPS; kDa at the top of the chromatogram, molecular weights of the marker proteins). The recombinant PECPS was overexpressed in *E. coli* as a fusion protein with a hexahistidine tag at the N-terminus. The purified recombinant PECPS migrated as a single band with a molecular weight of 40 kDa on 12% (w/v) SDS–PAGE, in agreement with the calculated molecular weight of 43 kDa. In contrast, a size-exclusion chromatography indicated a molecular weight of about 86 kDa. Each experiment was repeated for three times.

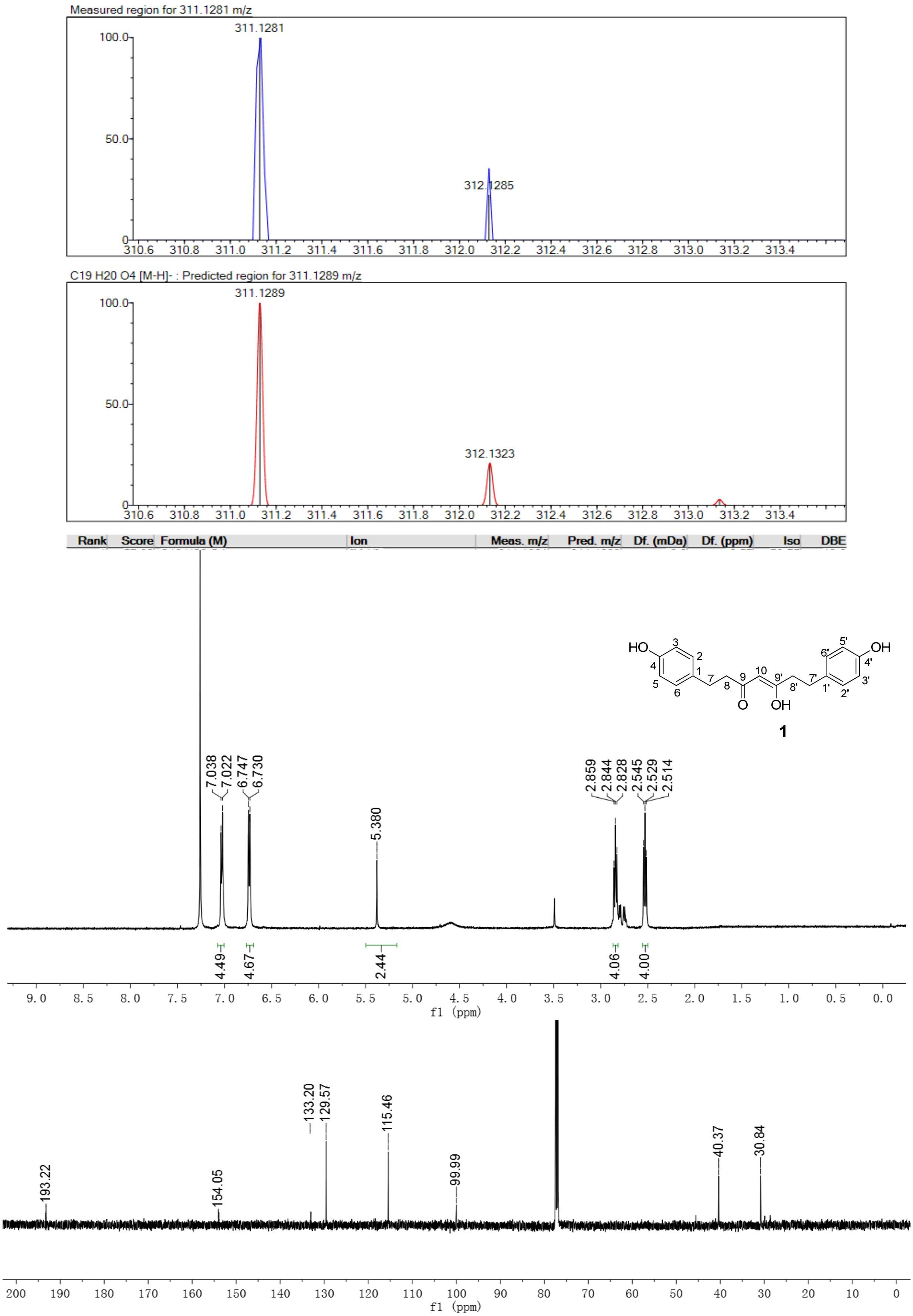

Figure 5. The HRESI MS, ^{1}H NMR, and ^{13}C NMR spectra of 1 in $CDCl_3$

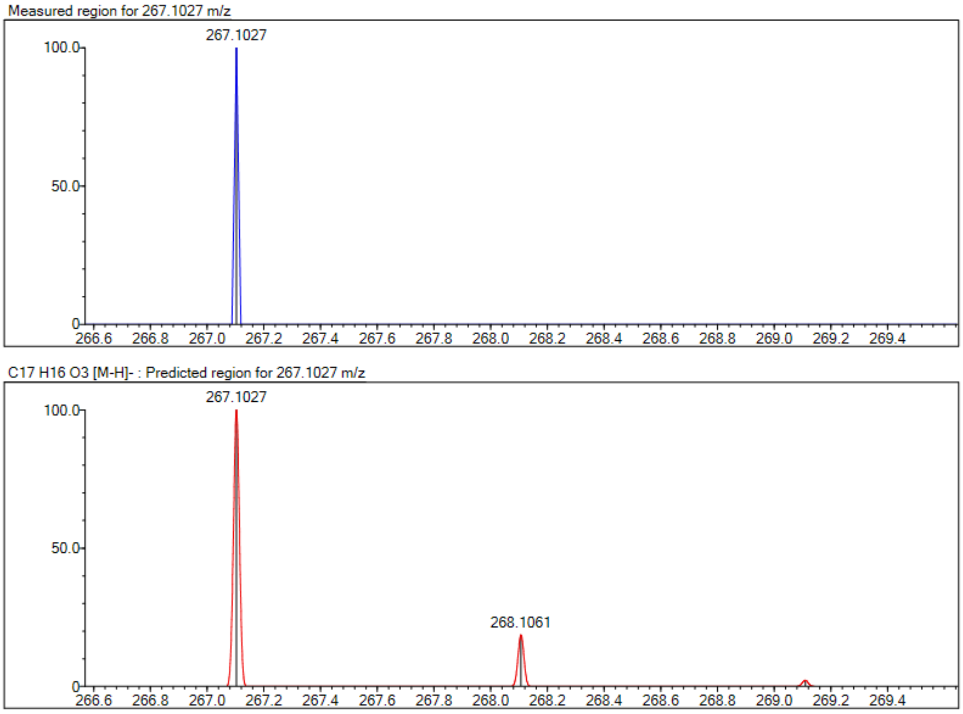

Rank	Score	Formula (M)	Ion	Meas. m/z	Pred. m/z	Df. (mDa)	Df. (ppm)	Iso	DBE
1	0.00	C17 H16 O3	[M-H]-	267.1027	267.1027	0.0	0.00	0.00	10.0

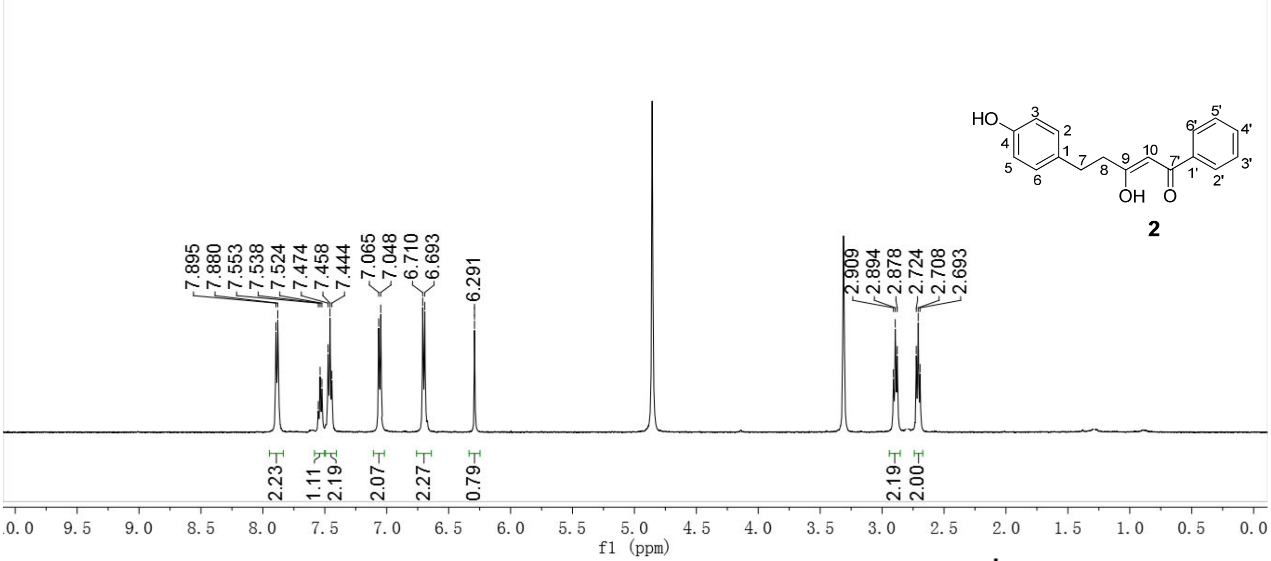

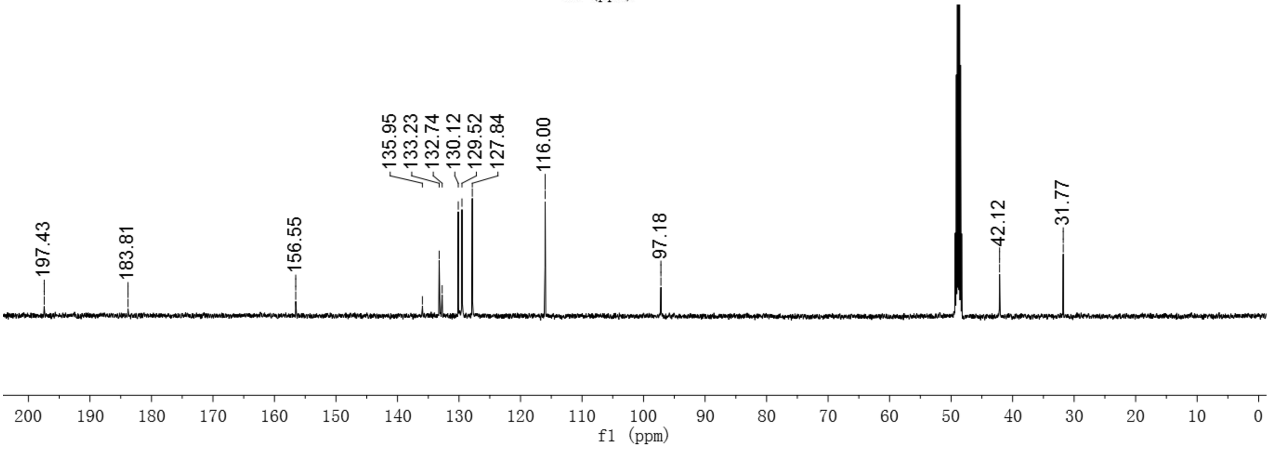

Figure 6. The HRESI MS, ^{1}H NMR, and ^{13}C NMR spectra of 2 in CD_3OD

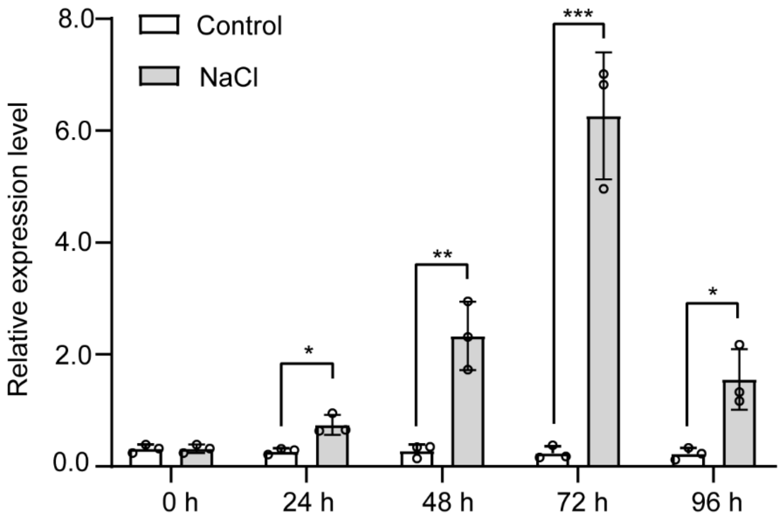

Figure 7. The expression levels of *PECPS* in NaCl-treated *A. sinenesis* calli at different time points. The healthy calli under the same conditions served as controls. Relative expression levels of *PECPS* were quantified by qPCR and normalized to the amount of *GAPDH* gene. Data represent the mean ± SD (n = 3). Statistical significance was analyzed using two-tailed unpaired Student's t-tests. ***indicates $p< 0.001$; ** indicates $p< 0.01$; * indicates $p<0.05$. The exact *p*-values and source data are reported in the Source data file.

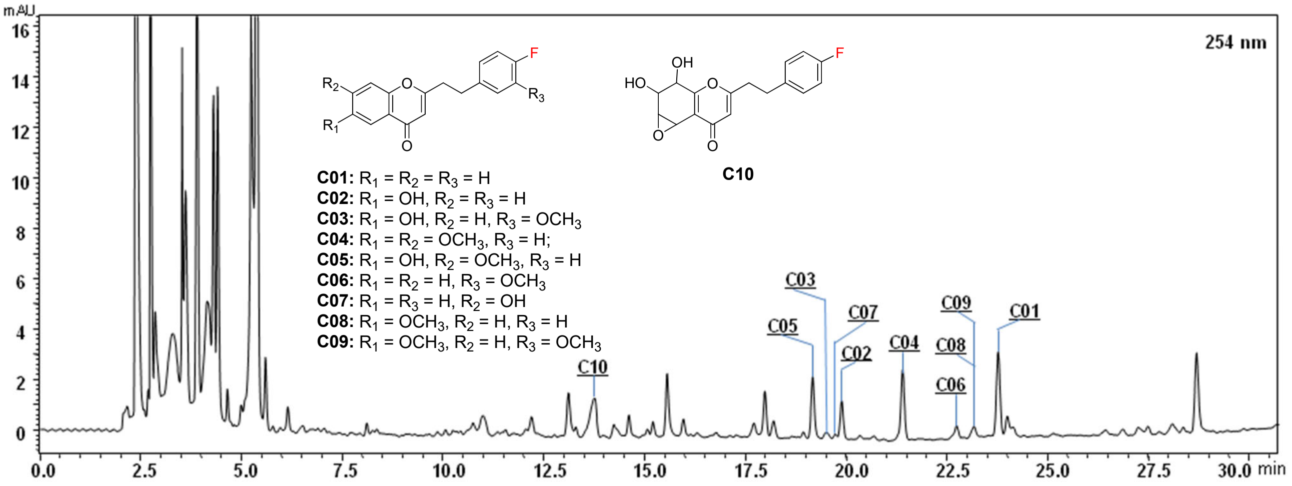

Figure 8. Fluorinated PECs in the crude extracts of suspension cells fed 5-(4-F-phenyl)-1-phenylpentane-1,3-dione (**F-2**). The HPLC chromatogram (UV, 254 nm) was recorded on an Agilent Eclipse XDB C_{18} column (250 mm × 4.6 mm, I.D., 5 μm) eluted with H_2O and acetonitrile in a gradient mode (0–30 min, 5%–95% acetonitrile; flow rate 1.0 mL/min). Note: Only compounds (**C01-C10**) that were finally purified and elucidated from the crude extract are marked in the chromatogram, and the other peaks are not labeled because their structures were not determined.

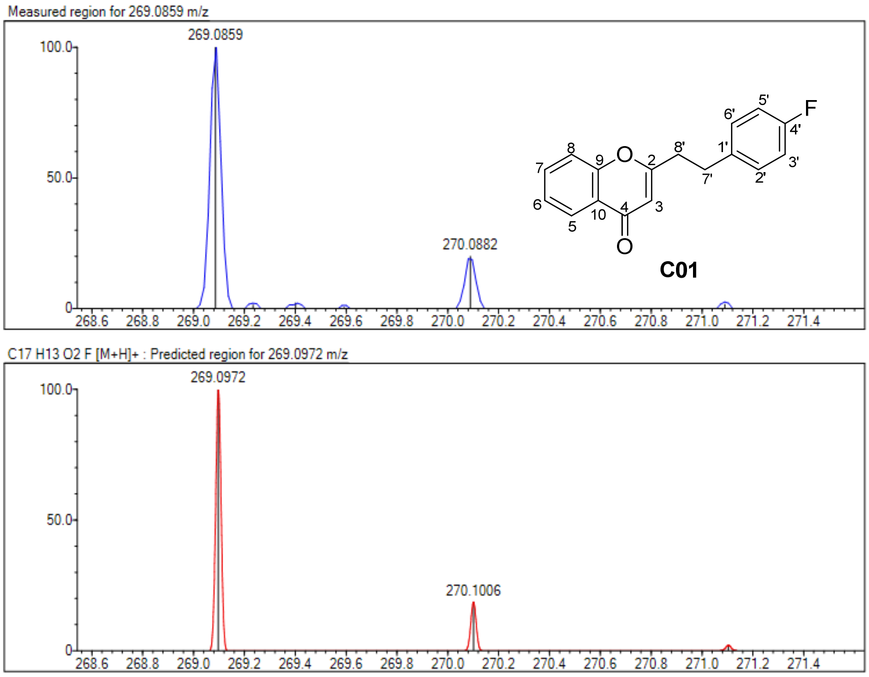

Rank	Score	Formula (M)	Ion	Meas. m/z	Pred. m/z	Df. (mDa)	Df. (ppm)	Iso	DBE
5	0.00	C17 H13 O2 F	[M+H]+	269.0859	269.0972	-11.3	-41.99	25.51	11.0

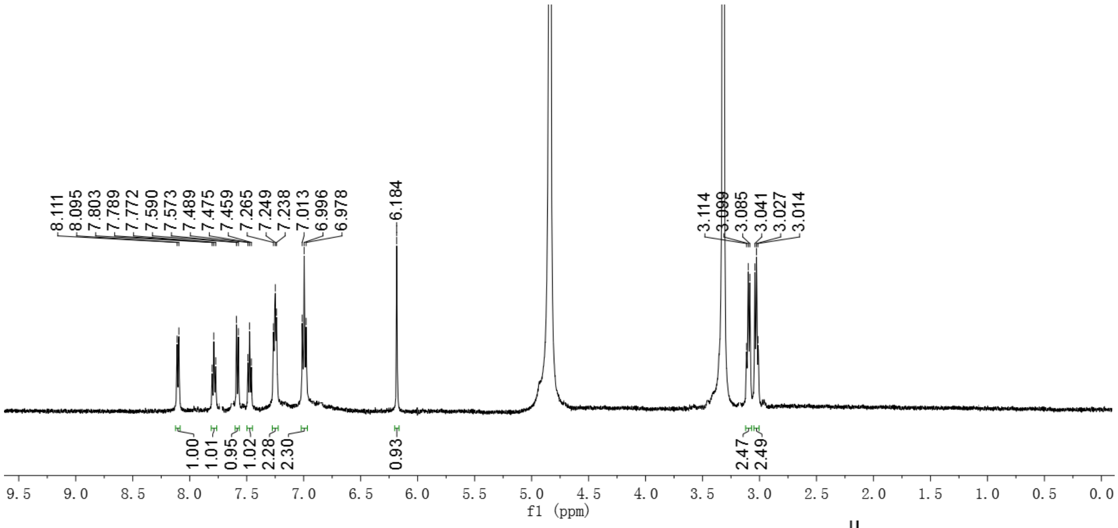

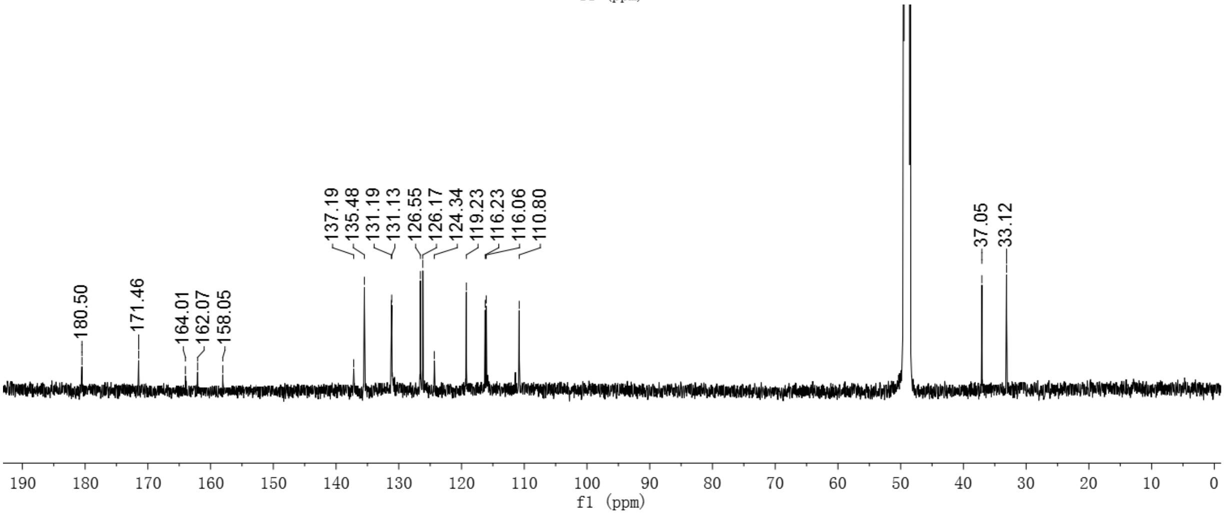

Figure 9. The HRESI MS, ^{1}H NMR, and ^{13}C NMR spectra of C01 in CD_3OD

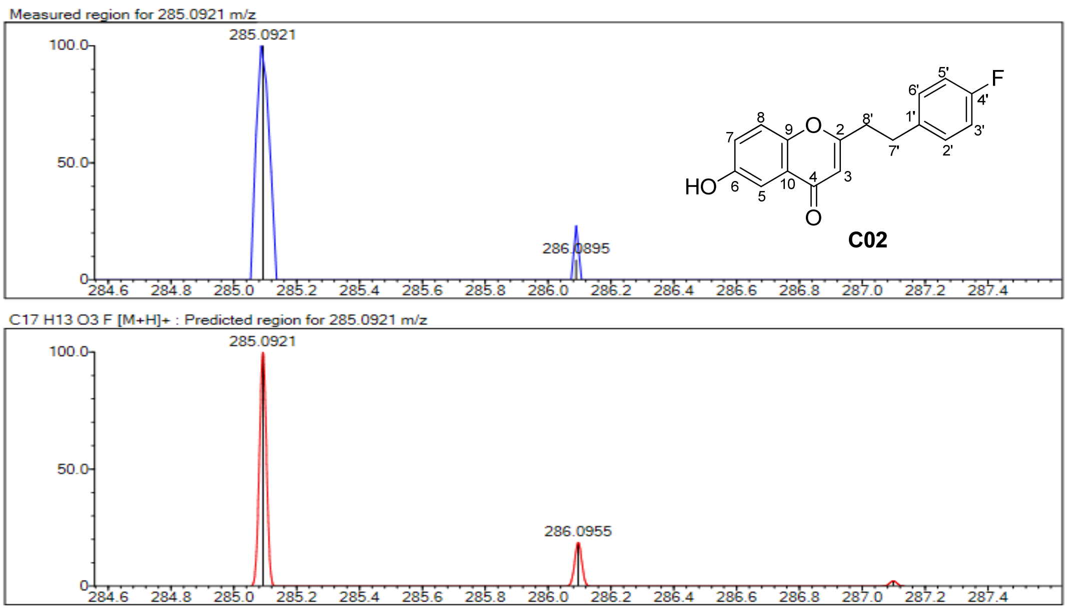

Rank	Score	Formula (M)	Ion	Meas. m/z	Pred. m/z	Df. (mDa)	Df. (ppm)	Iso	DBE
1	49.15	C17 H13 O3 F	[M+H]+	285.0921	285.0921	-0.0	0.00	49.15	11.0

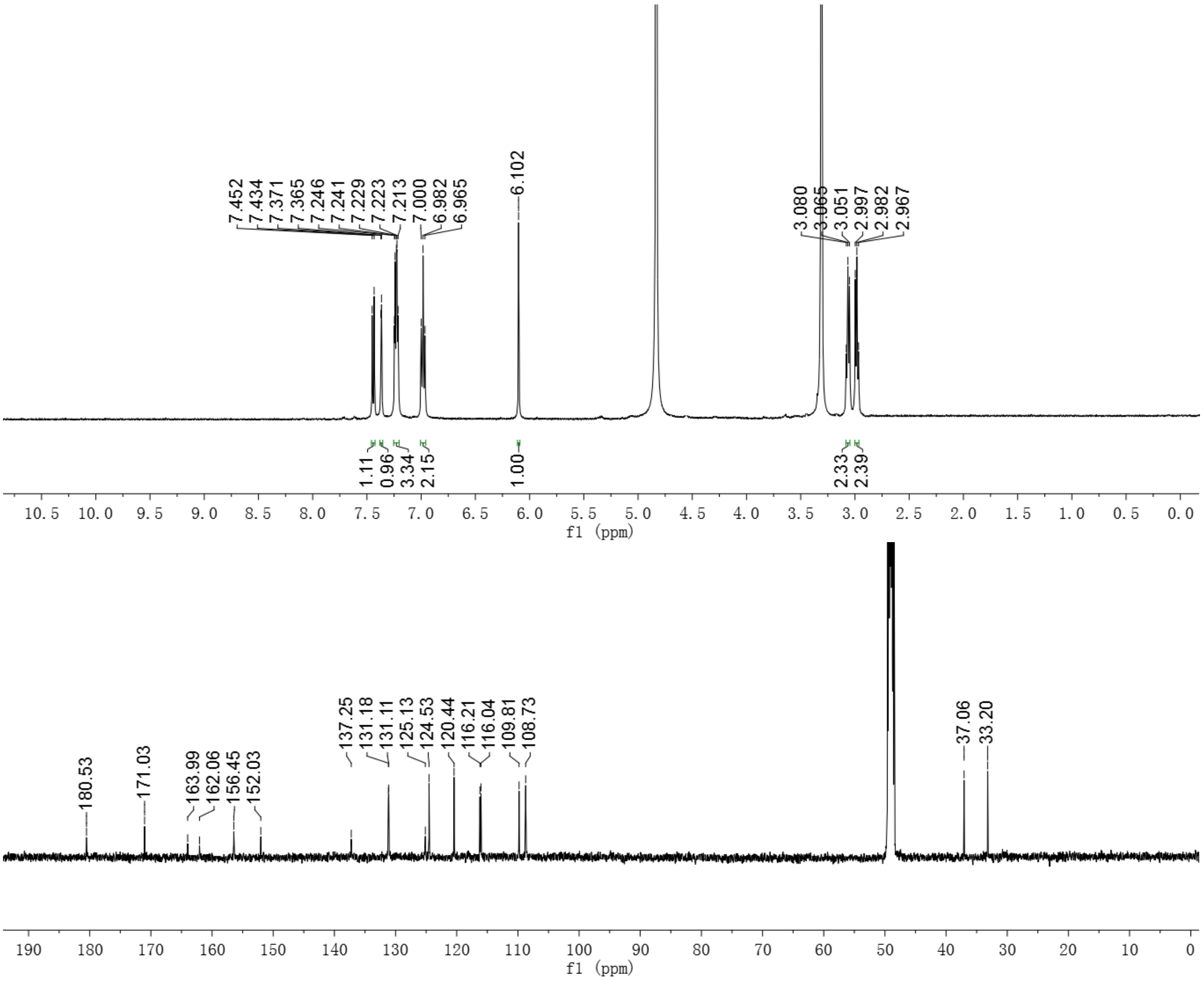

Figure 10. The HRESI MS, ^{1}H NMR, and ^{13}C NMR spectra of C02 in CD_3OD

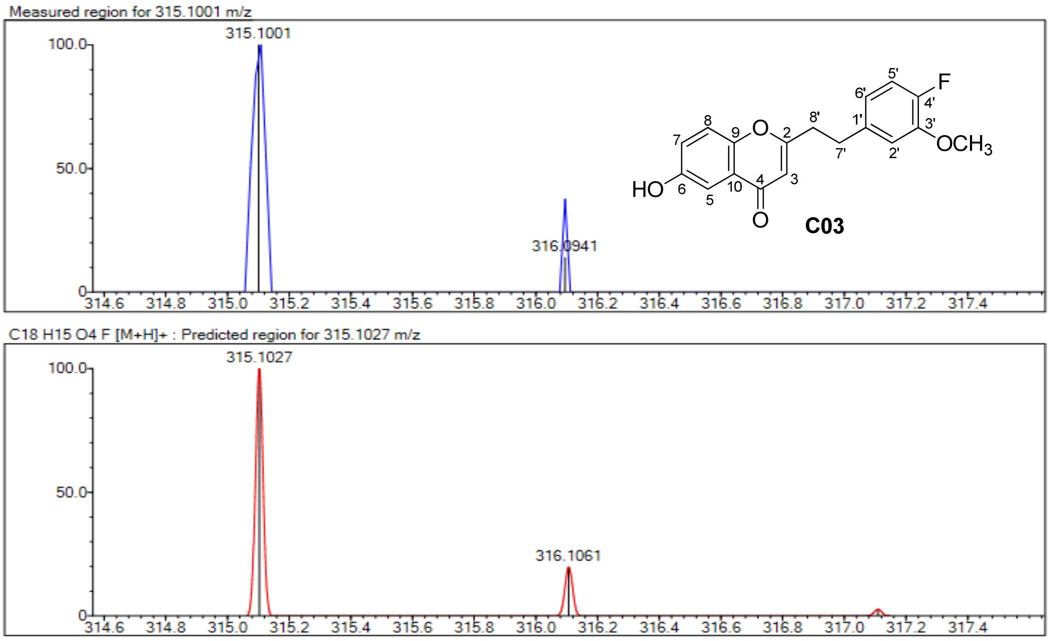

Rank	Score	Formula (M)	Ion	Meas. m/z	Pred. m/z	Df. (mDa)	Df. (ppm)	Iso	DBE
1	17.75	C18 H15 O4 F	[M+H]+	315.1001	315.1027	-2.6	-8.25	30.87	11.0

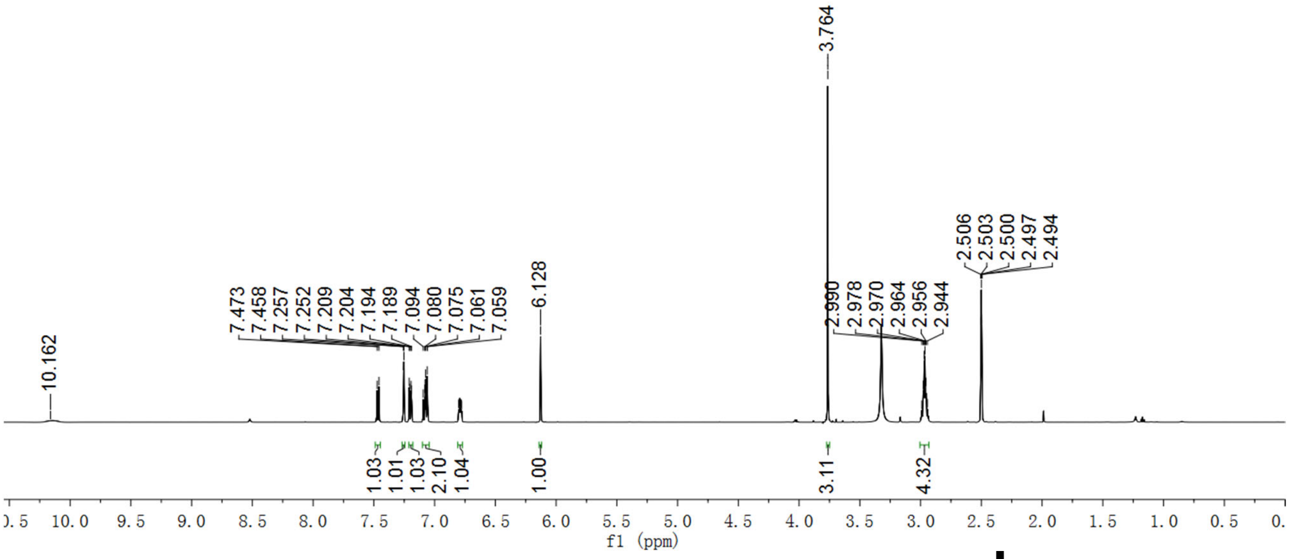

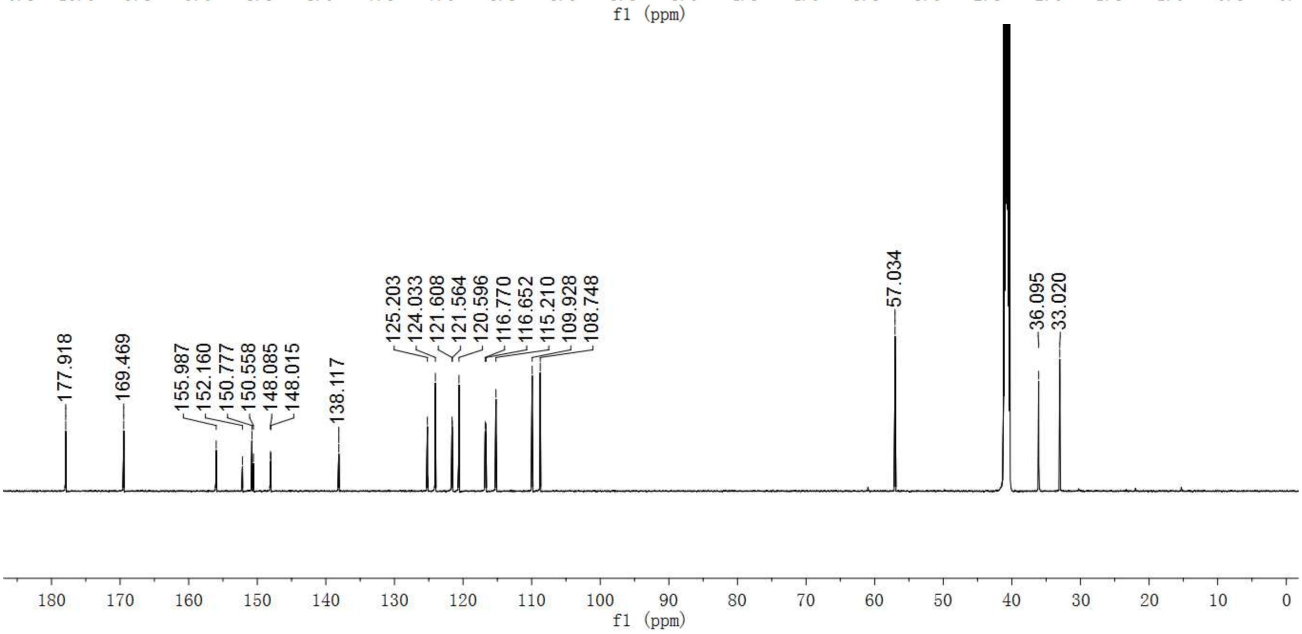

Figure 11. The HRESI MS, ^{1}H NMR, and ^{13}C NMR spectra of C03 in DMSO-*d₆*

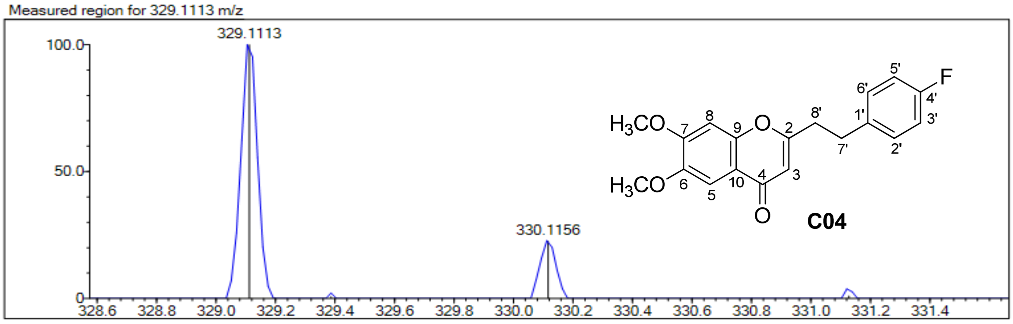

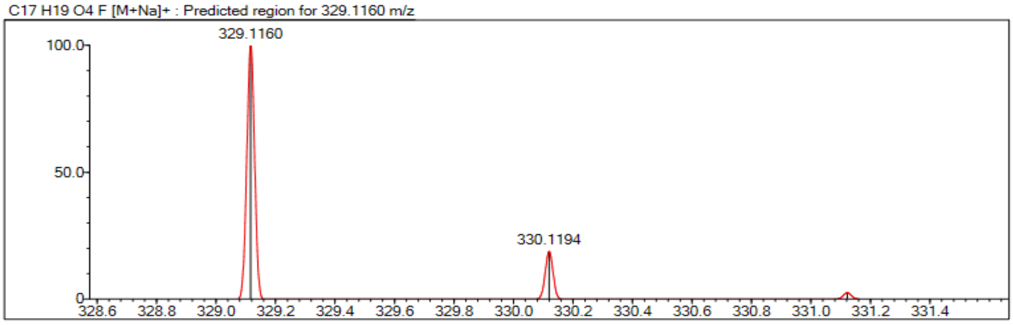

Rank	Score	Formula (M)	Ion	Meas. m/z	Pred. m/z	Df. (mDa)	Df. (ppm)	Iso	DBE
1	22.17	C17 H19 O4 F	[M+Na]+	329.1113	329.1160	-4.7	-14.28	77.57	8.0

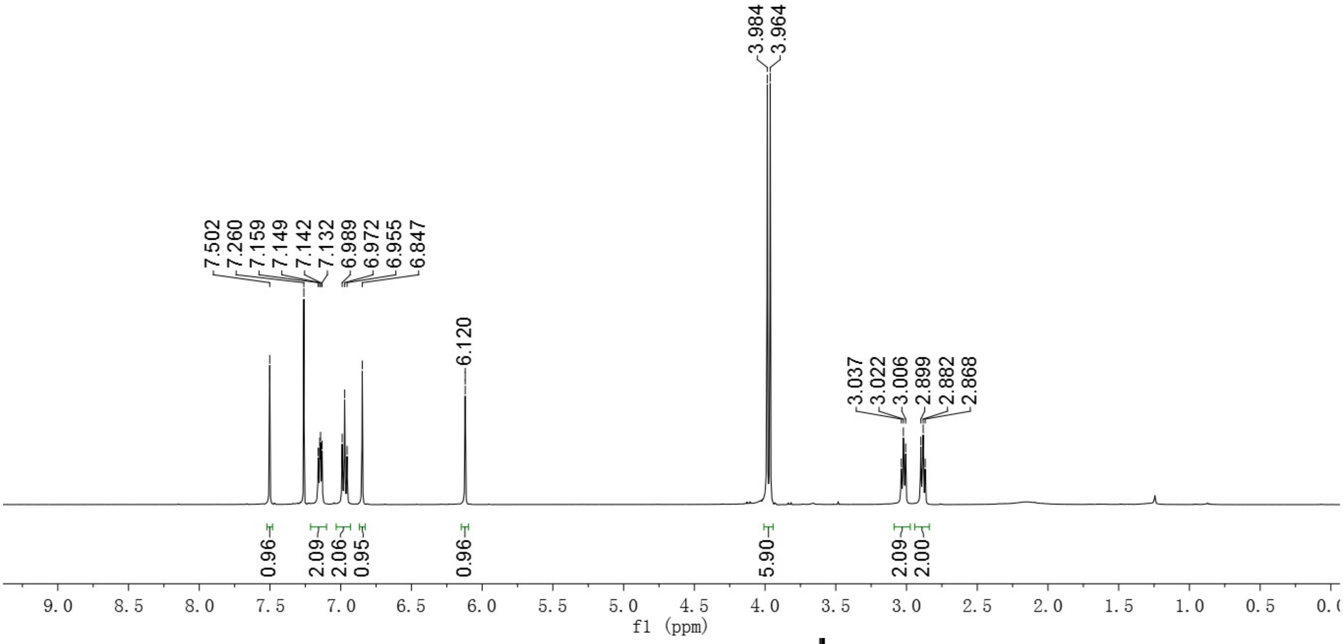

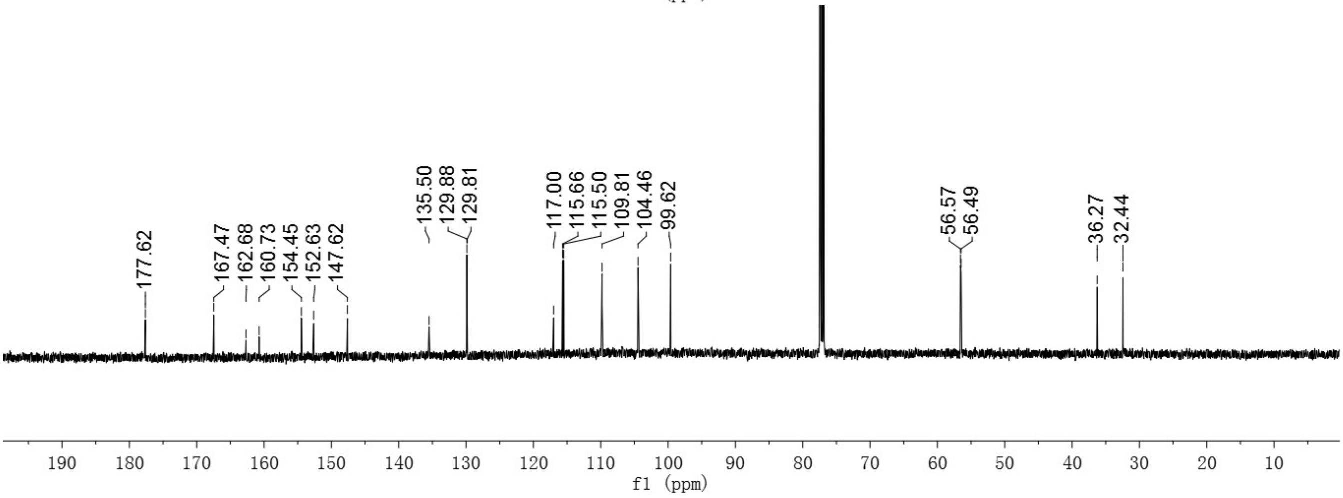

Figure 12. The HRESI MS, ^{1}H NMR, and ^{13}C NMR spectra of C04 in $CDCl_3$

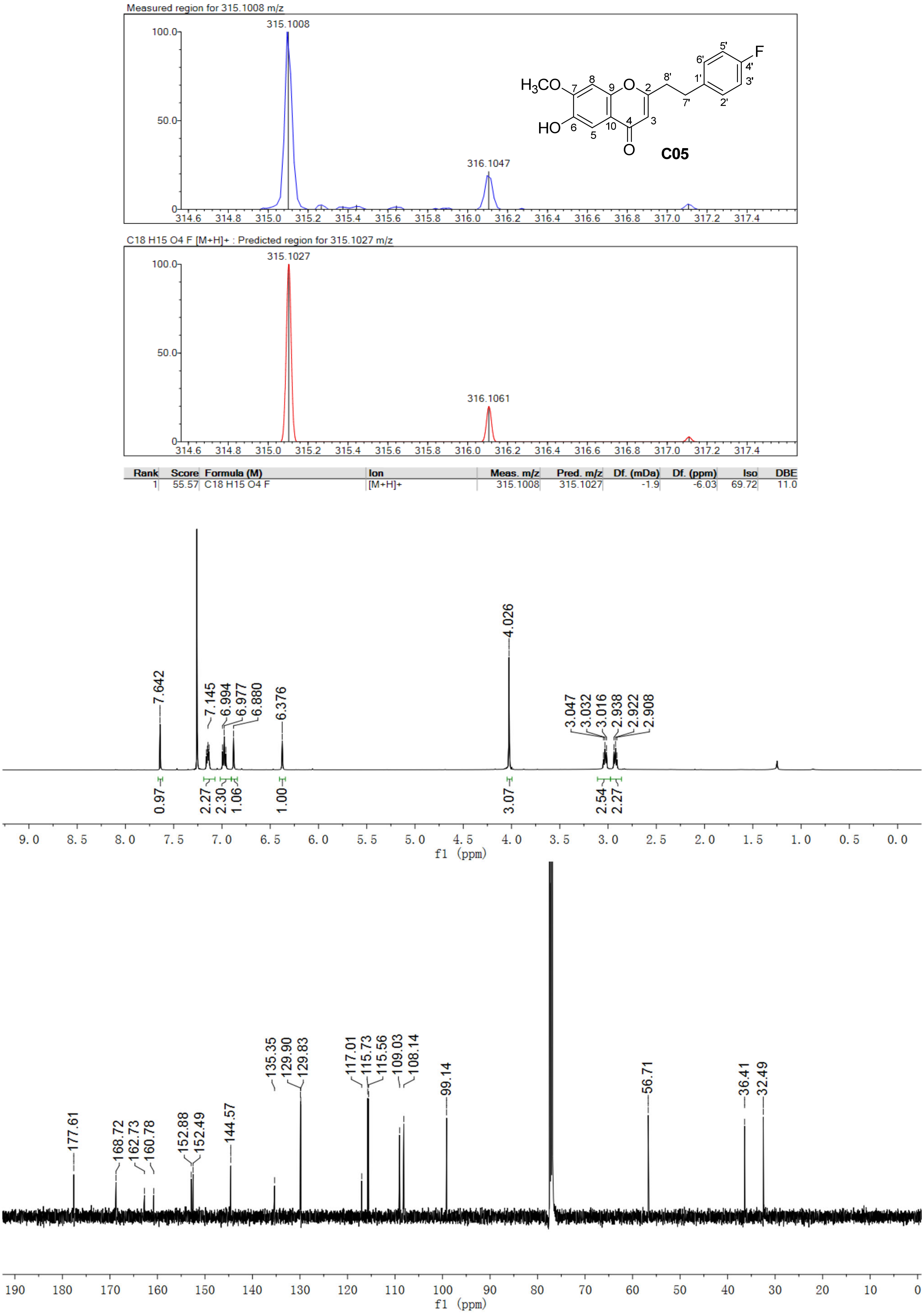

Figure 13. The HRESI MS, ^{1}H NMR, and ^{13}C NMR spectra of C05 in CDCl$_3$

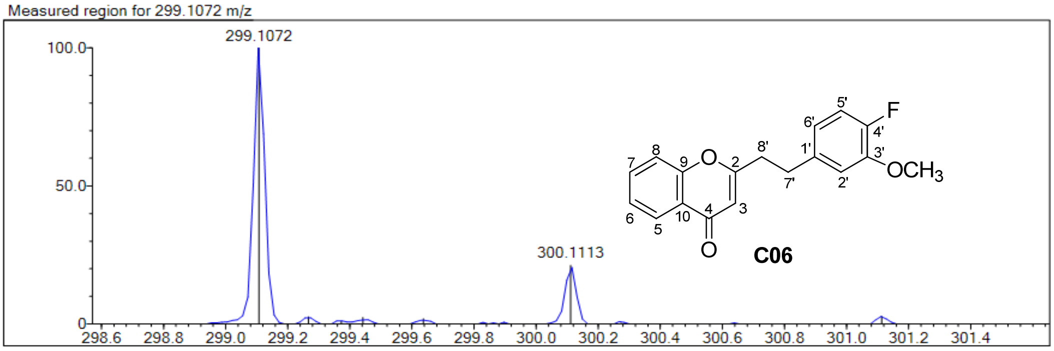

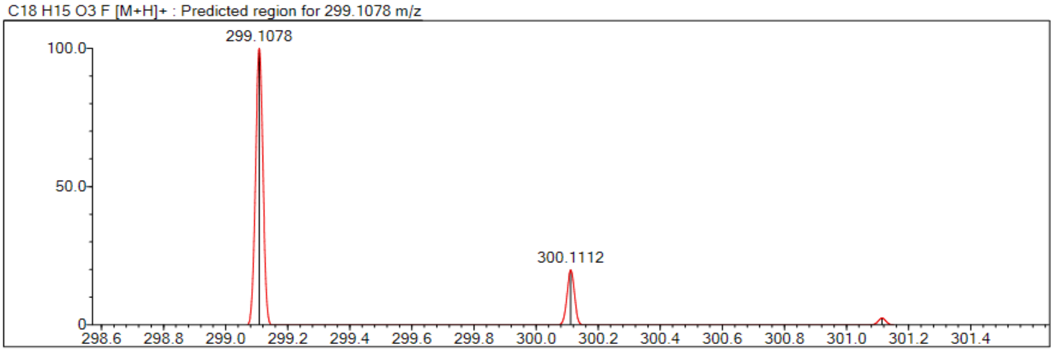

Rank	Score	Formula (M)	Ion	Meas. m/z	Pred. m/z	Df. (mDa)	Df. (ppm)	Iso	DBE
1	89.74	C18 H15 O3 F	[M+H]+	299.1072	299.1078	-0.6	-2.01	92.06	11.0

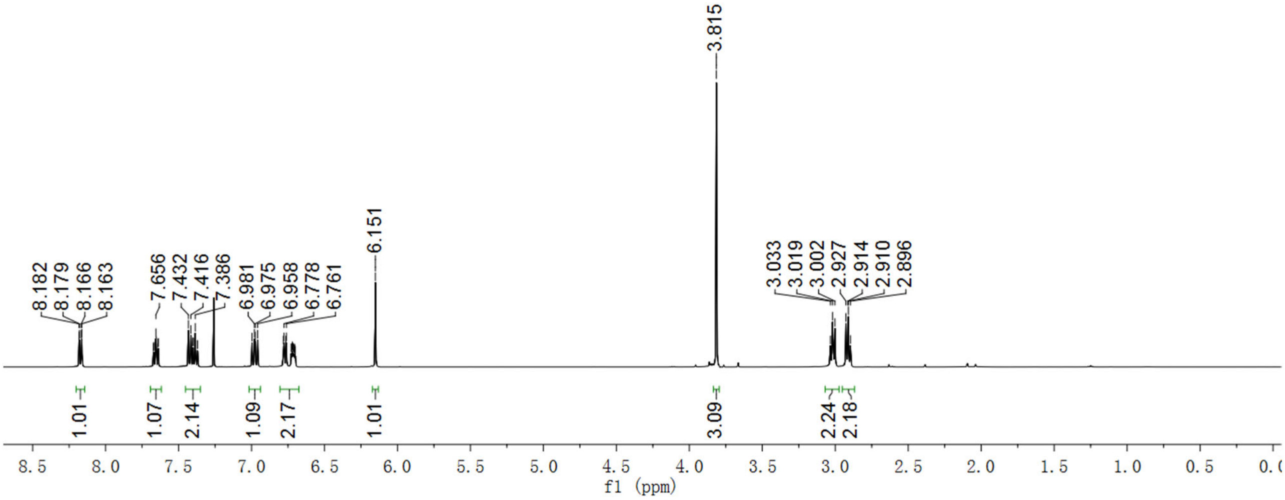

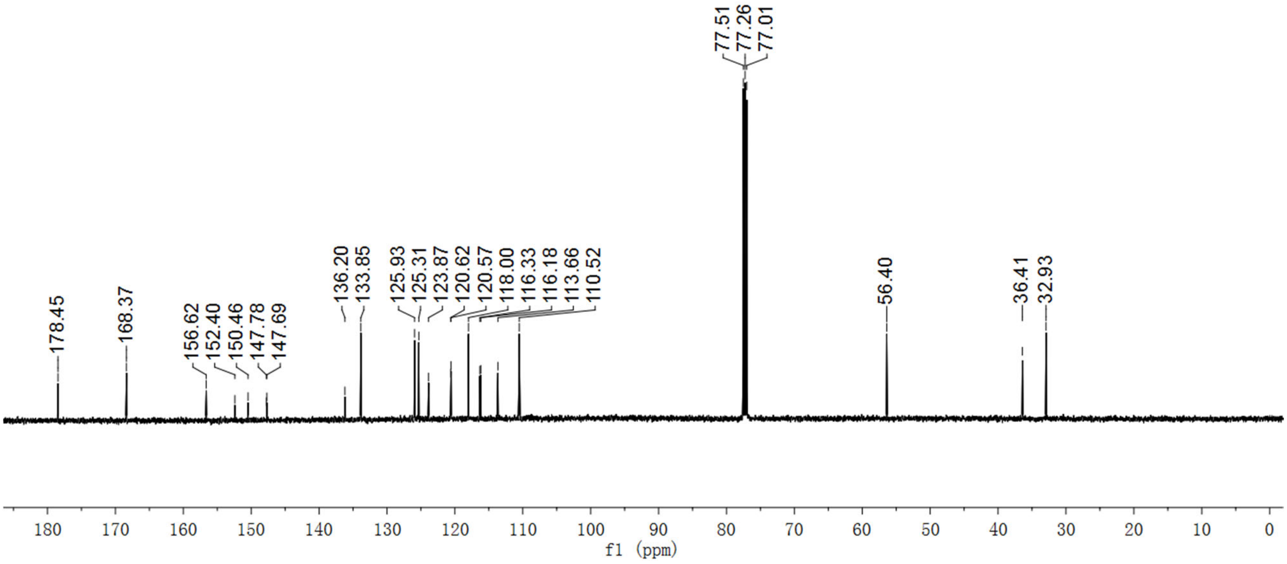

Figure 14. The HRESI MS, ^{1}H NMR, and ^{13}C NMR spectra of C06 in $CDCl_3$

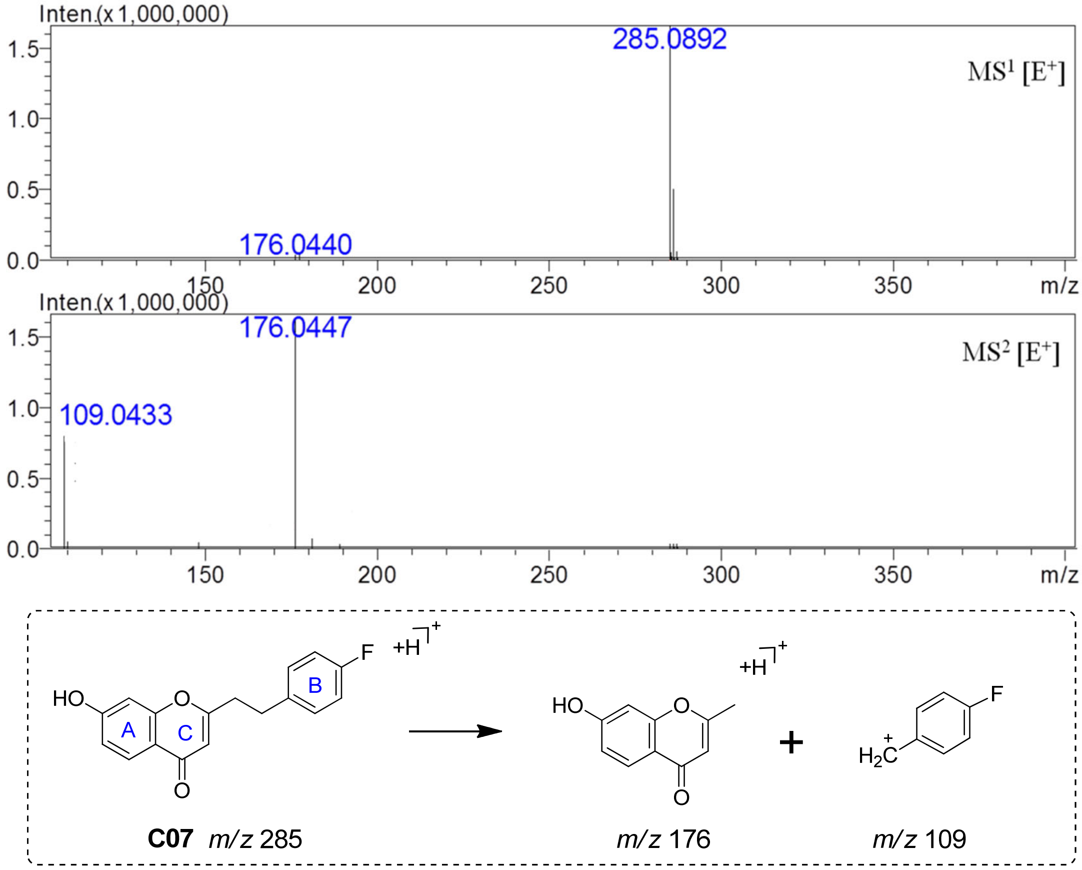

Figure 15. MS spectra (upper: MS^1, middle: MS^2) and proposed cleavage pathway (bottom) of C07. Note: The presence of an $[M + H]^+$ ion peak at *m/z* 285.0892 in the MS^1 spectrum allowed the assignment of the molecular formula of $C_{17}H_{13}FO_3$ (calc. for $C_{17}H_{14}FO_3$, *m/z* 285.0927). The two characteristic fragments at *m/z* 109.0433 and *m/z* 176.0447 observed in the MS^2 spectrum suggested that **C07** is a fluorinated phenylethylchromone with a hydroxyl group on the left A ring. Although the MS data did not allow the exact position of the hydroxyl group to be determined, considering that naturally occurring phenylethylchromones in agarwood often bear hydroxyl groups at C-6 and C-7, and the C-6 hydroxylated phenylethyl chromone (**C02**) has already been unambiguously elucidated by NMR, the substituted position of the hydroxyl group was tentatively assigned to C-7.

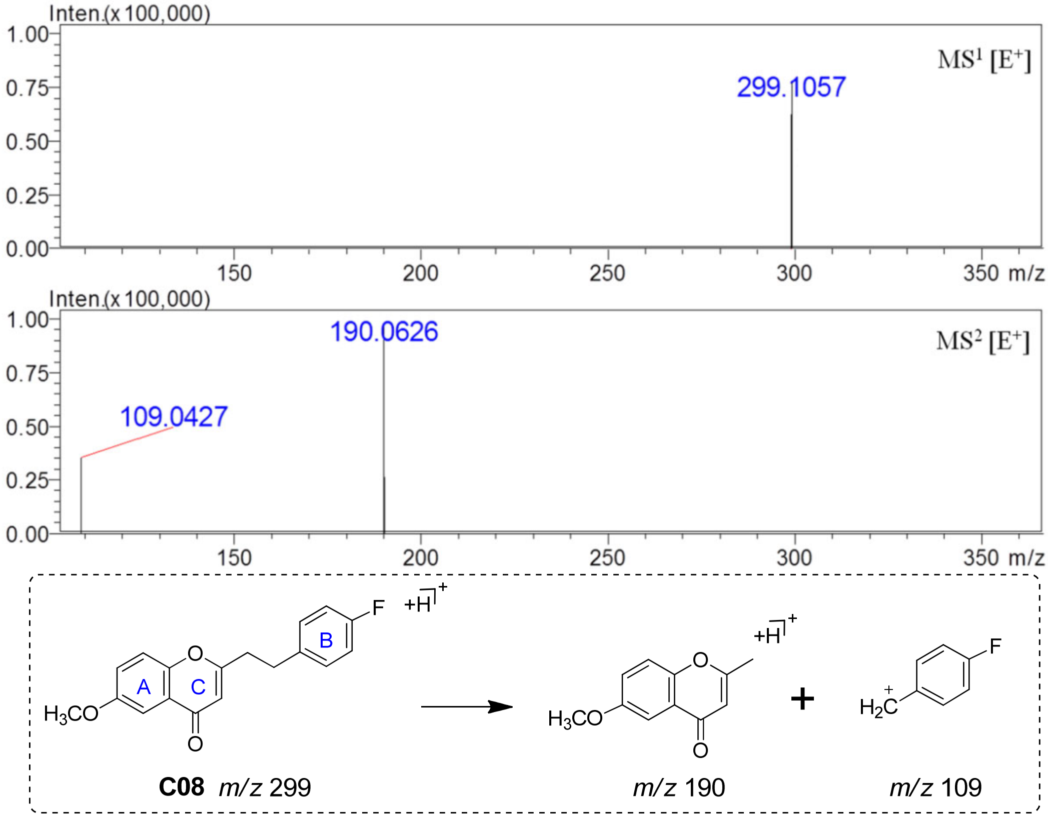

Figure 16. MS spectra (upper: MS^1, middle: MS^2) and proposed cleavage pathway (bottom) of C08. Note: The presence of an $[M + H]^+$ ion peak at *m/z* 299.1057 in the MS^1 spectrum allowed the assignment of the molecular formula of $C_{18}H_{15}FO_3$ (calc. for $C_{18}H_{16}FO_3$, *m/z* 299.1083). The two characteristic fragments at *m/z* 109.0427 and *m/z* 190.0626 observed in the MS^2 spectrum suggested that **C08** is a fluorinated phenylethyl chromone with a methoxy group on the left A ring. Considering that 6-methoxyphenylethylchromones from agarwood are often reported, here we tentatively assigned the methoxy group to C-6.

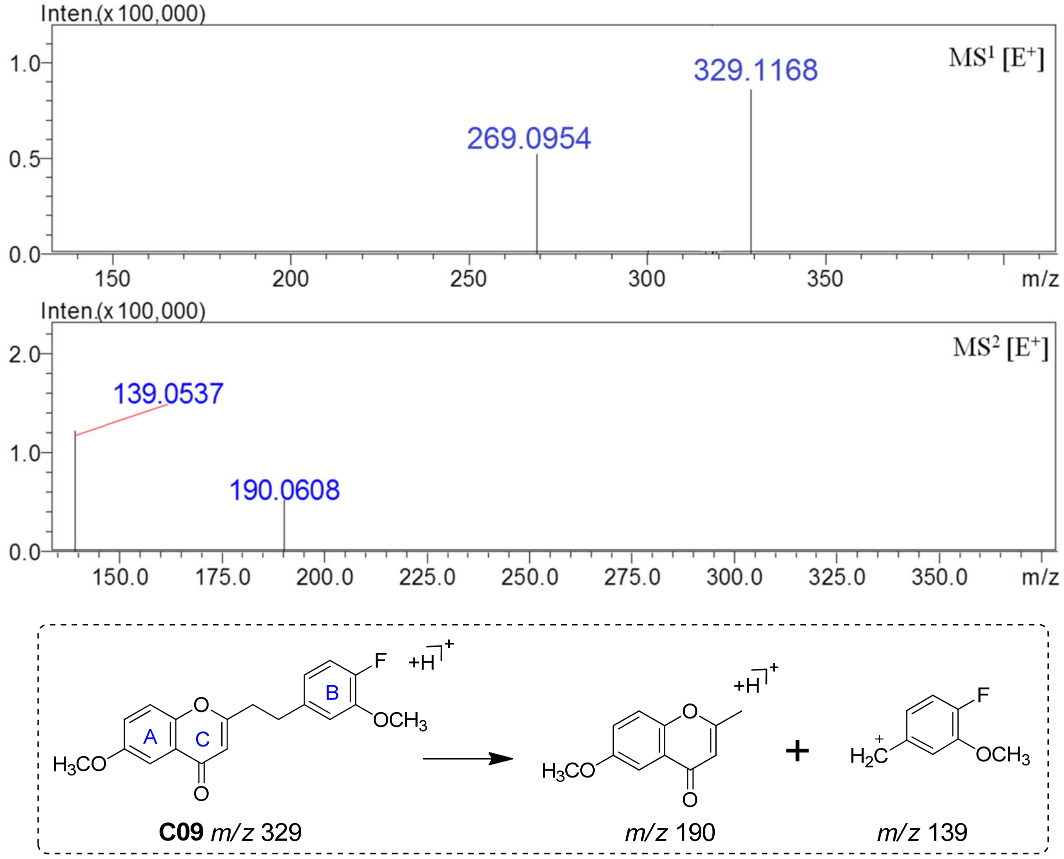

Figure 17. MS spectra (upper: MS^1**, middle: MS**^{2}**) and proposed cleavage pathway (bottom) of C09.** Note: The presence of an $[M + H]^+$ ion peak at *m/z* 329.1168 in the MS^1 spectrum allowed the assignment of the molecular formula of $C_{19}H_{17}FO_4$ (calc. for $C_{19}H_{18}FO_4$, *m/z* 329.1189). The two characteristic fragments at *m/z* 139.0537 and *m/z* 190.0608 observed in the MS^2 spectrum suggested that **C09** is a fluorinated phenylethylchromone with one methoxy group on the left A-ring and one methoxy group on the right B ring, respectively. Considering that 6-hydroxy-(4-fluoro-3-methoxyphenylethyl)chromone (**C03**) has been unambiguously determined, the two methoxy groups were assigned to C-6 and C-3', respectively.

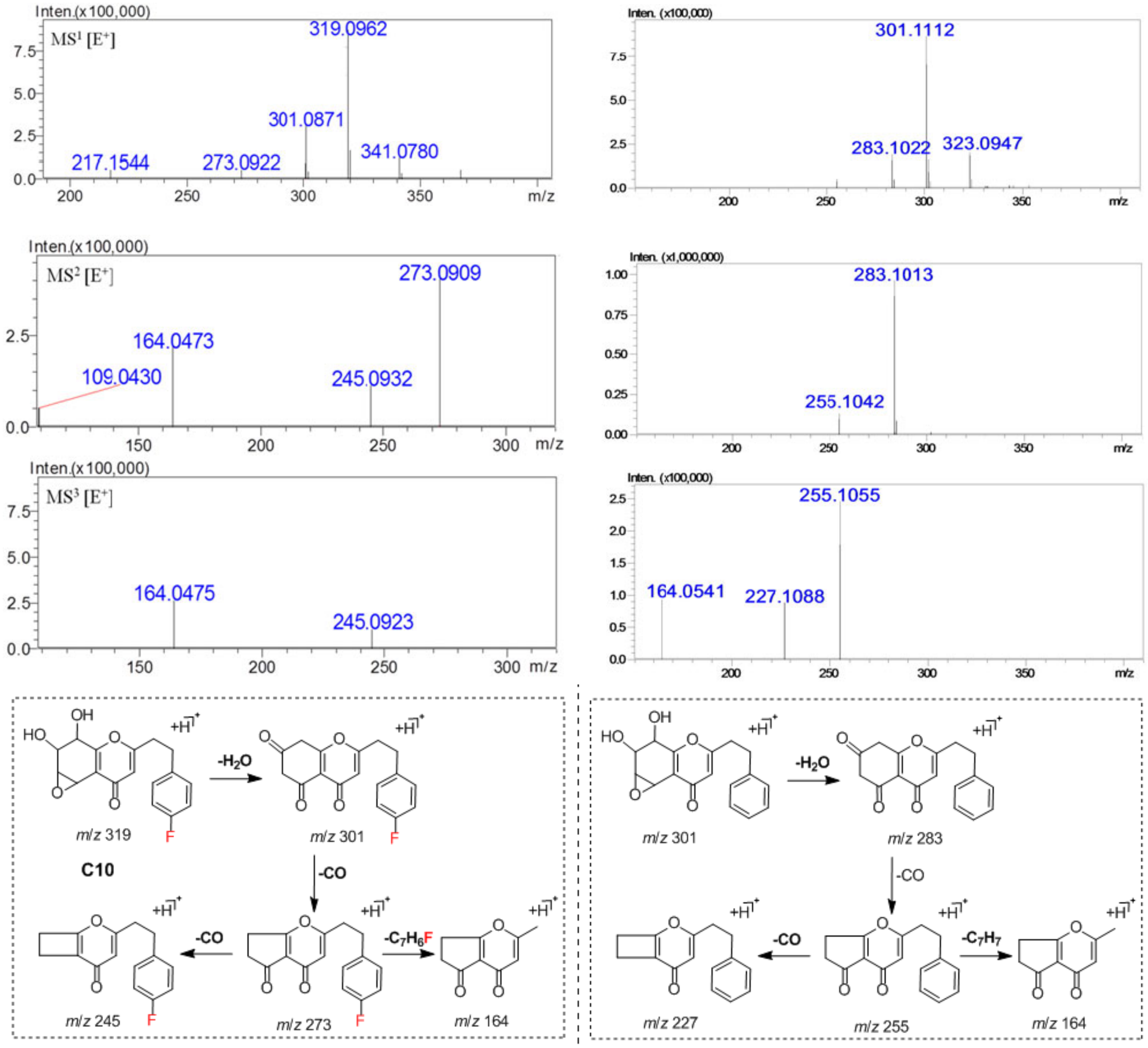

Figure 18. MS spectra and proposed cleavage pathway of C10 (left column) and its analogue previously isolated from agarwood (right column). Note: The presence of an $[M + H]^+$ ion peak at *m/z* 319.0962 (calc. for $C_{17}H_{16}FO_5$, *m/z* 319.0982) and an $[M + Na]^+$ ion peak at *m/z* 341.0780 (calc. for $C_{17}H_{15}FO_5Na$, *m/z* 341.0801) in the MS^1 spectrum allowed the assignment of the molecular formula of $C_{17}H_{15}FO_5$, suggesting that **C10** is a highly oxygenated, fluorinated phenylethylchromone. Comparison of the MS data of **C10** (left column) with those of its analogue previously isolated from agarwood (right column) revealed that these two compounds undergo the completely identical cleavage mode to give the characteristic fragments $[M – H_2O + H]^+$, $[M – H_2O – CO + H]^+$, $[M – H_2O – CO – CO + H]^+$, and the same fragment at *m/z* 164, suggesting that these two compounds share a similar skeleton. Thus, the structure of **C10** was tentatively assigned as shown.

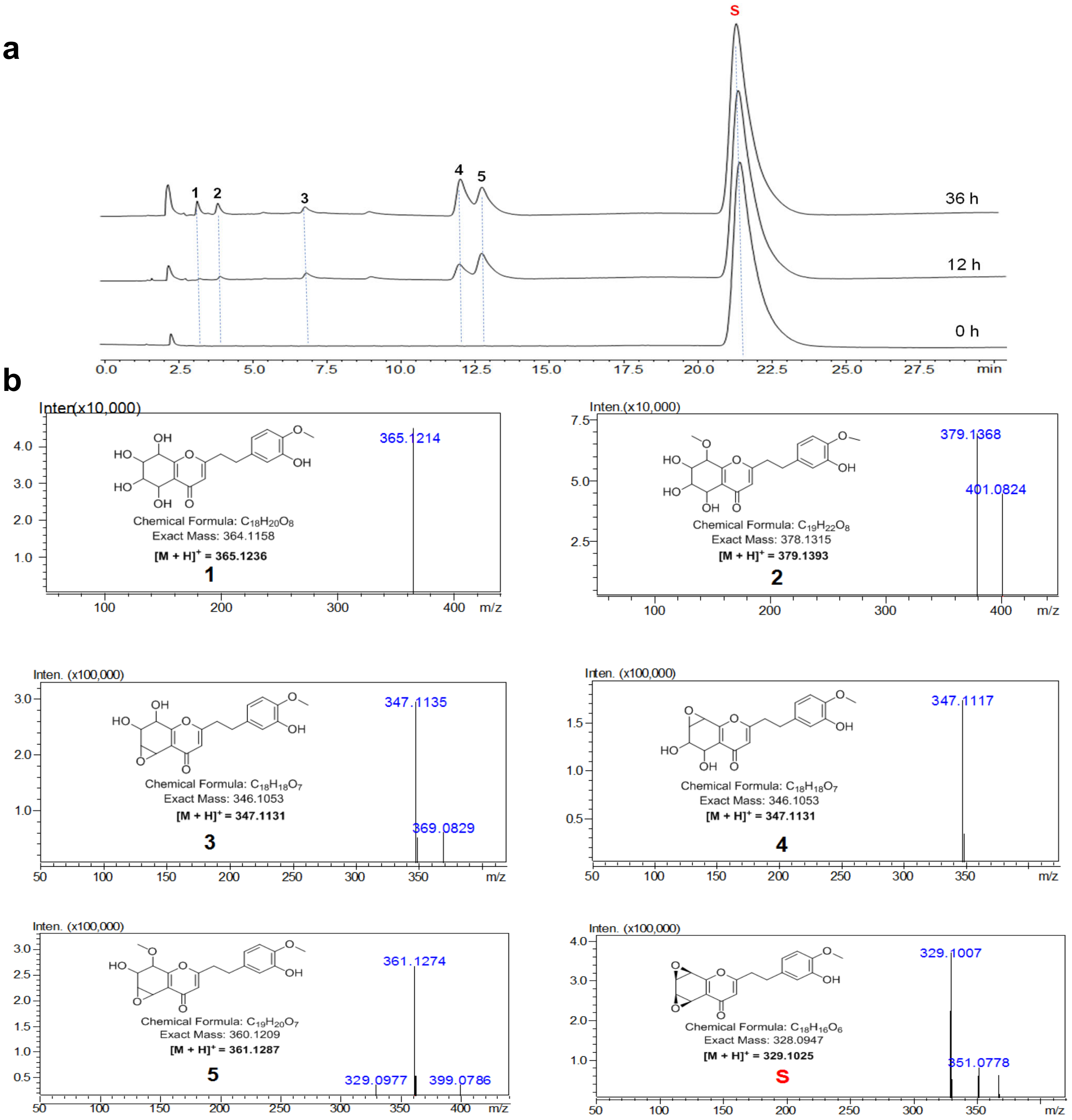

Figure 19. Non-enzymatic conversion of an OAC-type PEC, oxidoagarochromone C (S), to ATC-type PECs (1–5). a HPLC chromatograms (254 nm) obtained at 0 h, 12 h, and 36 h. **b** HRESI MS spectra of the substrate (**S**) and products (**1**–**5**). HPLC chromatograms were recorded on an Agilent ZORBAXSB C_{18} column (250 mm × 4.6 mm, I.D., 5 μm) eluted with H_2O and acetonitrile in an isocratic mode (0–30 min, 30% acetonitrile; flow rate 1.0 mL/min). Oxidoagarochromone C (**S**) was dissolved in 50% aqueous methanol and kept at room temperature. ATC-type PECs (**1**–**5**) converted from the substrate (**S**) *via* the non-enzymatic opening of the epoxy rings were readily apparent at 12 h and 36 h. Given that the structures of compounds **1-5** were tentatively identified by HRESI MS, the presence of methyl groups in compounds **2** and **5** is also possible on other hydroxyl groups formed by the opening of the epoxy rings, and the structures of **3** and **4** may be exchanged.

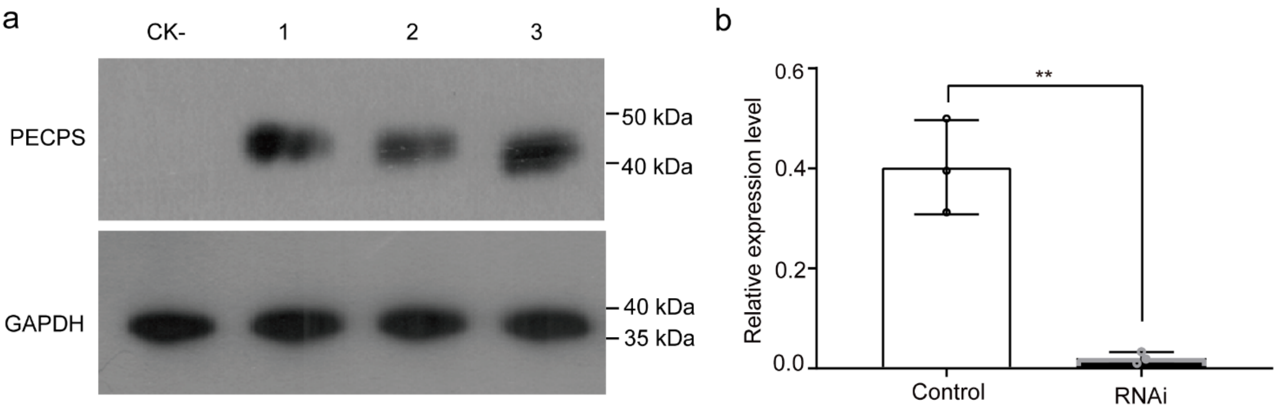

Figure 20. **Analysis of the expression of PECPS. a** Western blot analysis of the expression of PECPS in three different *N. benthamiana* leaves (from three plants) infiltrated with *Agrobacterium* harboring pCAMBIA1300-35S-*PECPS* plasmid, GAPDH was used as internal reference, and leaves infiltrated with *Agrobacterium* harboring pCAMBIA1300-35S vector was used as control (CK-). Three times repeated with similar results. **b** qPCR analysis of the expression level of *PECPS* in gene knocked down *A. sinenesis* calli. The experiment was repeated three times, and the expression of *PECPS* in healthy *A. sinenesis* calli was used as control. Data represent the mean ± SD (n = 3). Data represent the mean ± SD (n = 3). Statistical significance was analyzed using two-tailed unpaired Student's t-tests. ** indicates $p < 0.01$. The exact *p*-value and source data are reported in the Source data file.

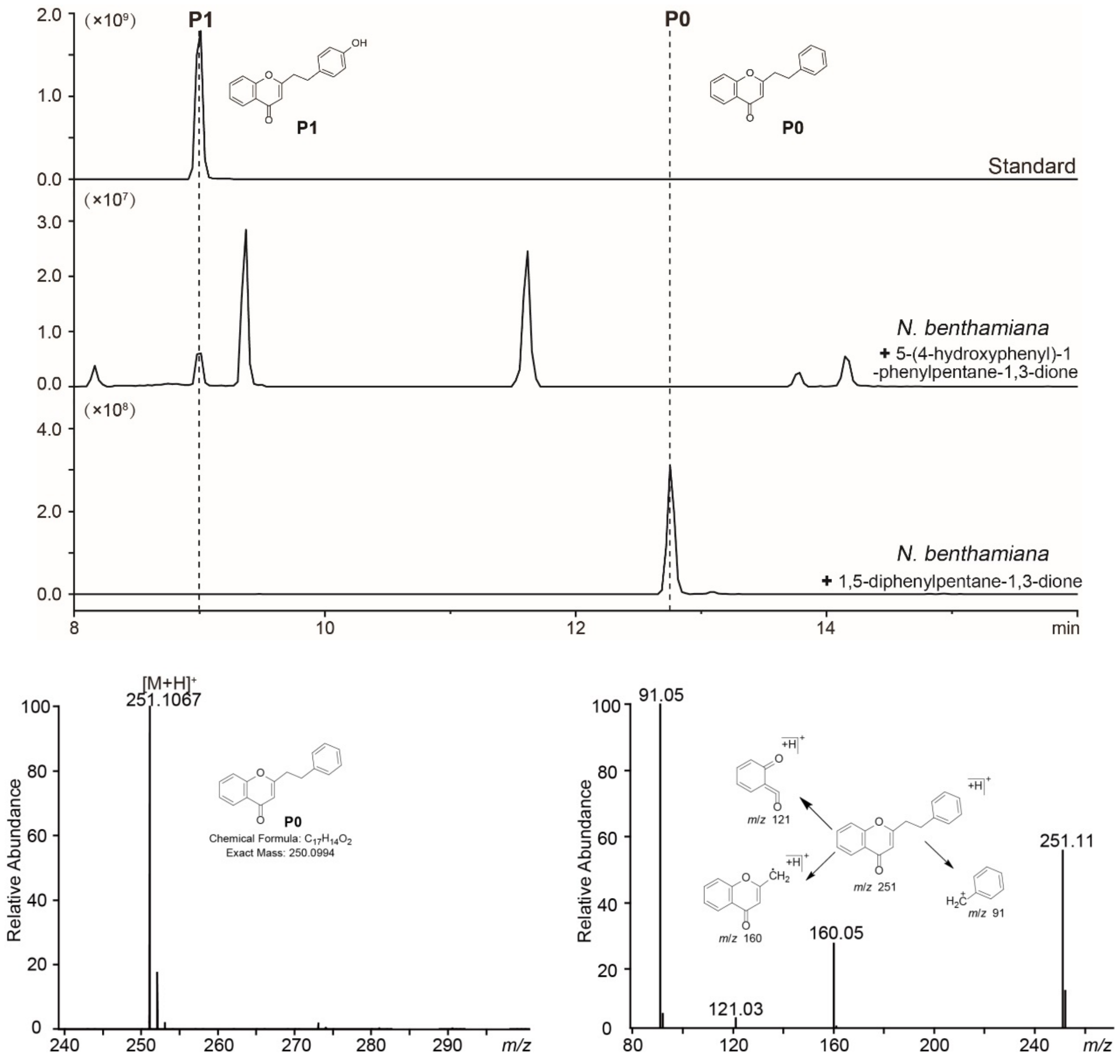

Figure 21. Transformation of the C_6-C_5-C_6 scaffolds into PECs by wild *N. benthamiana*. MS chromatogram (EIC) for the conversion of 5-(4-hydroxyphenyl)-1-phenylpentane-1,3-dione and 1,5-diphenylpentane-1,3-dione to 2-(4-hydroxyphenylethyl)-4*H*-chromen-4-one (**P1**) (the other peaks presented in the EIC chromatogram were not assigned because we could not deduce their structures based on the analysis of their MS and MS^2 spectra) and 2-phenylethyl-4*H*-chromen-4-one (**P0**) by wild type *N. benthamiana* and the MS spectra of **P0**. The structure of **P0** was tentatively assigned by careful analysis of its MS and MS^2 spectra.

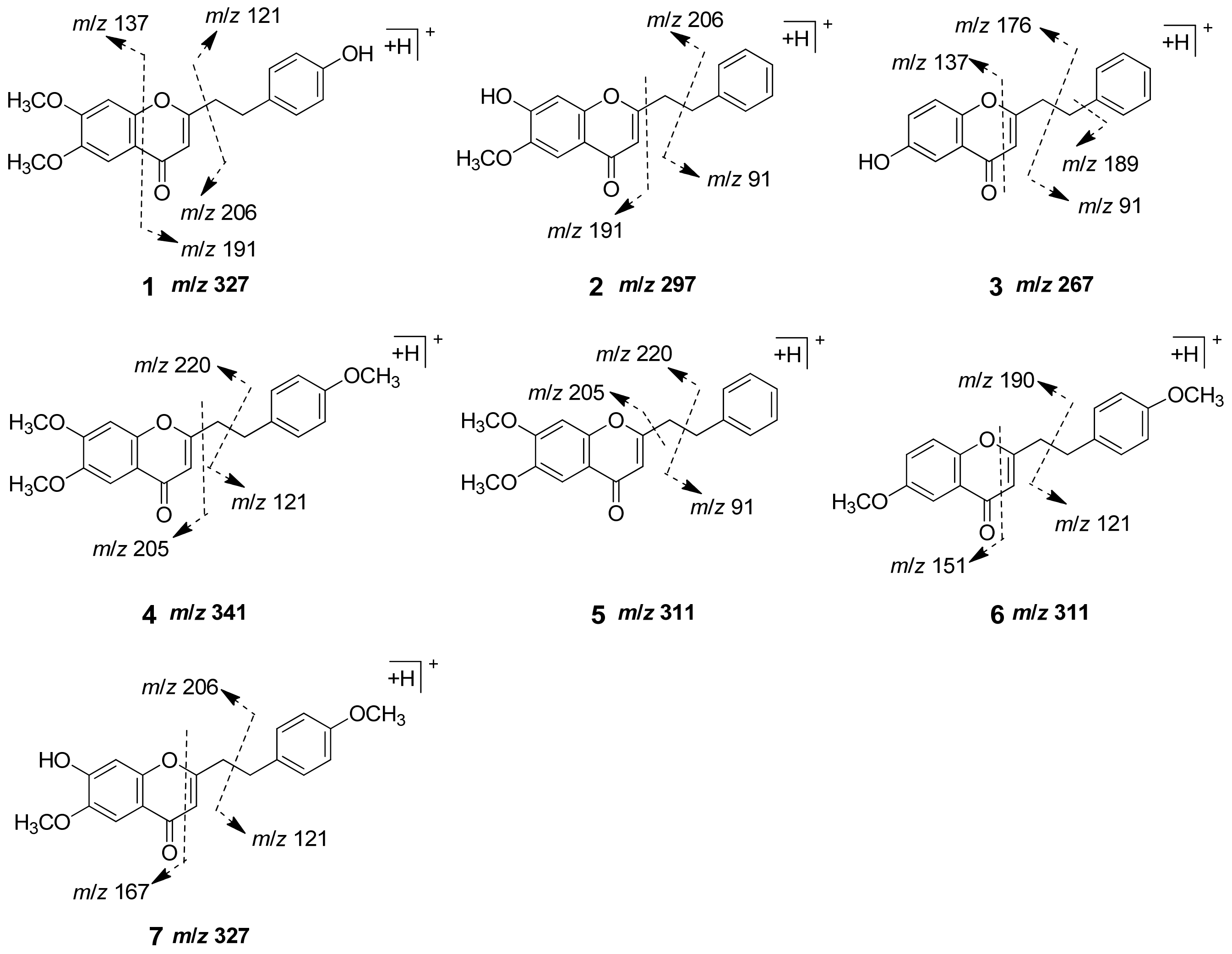

Figure 22. The structures of PECs 1–7 in NaCl-treated *PECPS* Knockdown *A. sinensis* calli. The major PECs **1**-**7** were tentatively assigned by their MS data recorded on Thermo Fisher Scientific high resolution Q Exactive Orbitrap mass spectrometer. Other PECs with tiny amount in *A. sinensis* calli were not presented here.

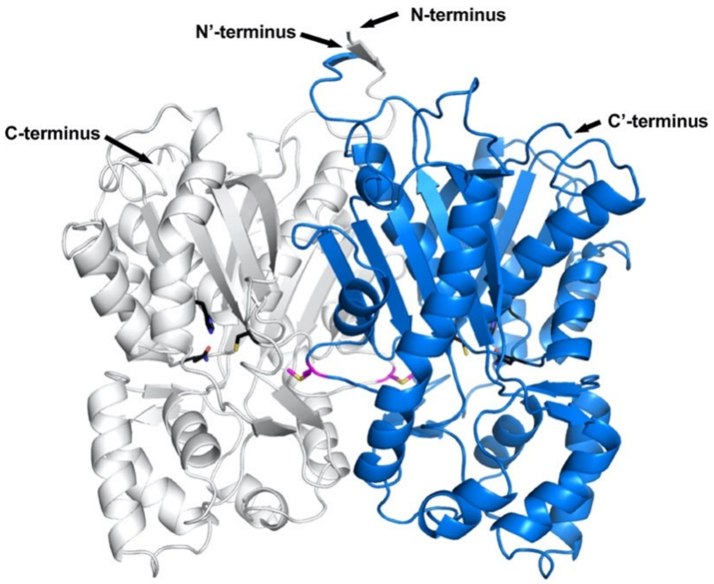

Figure 23. Overall homodimeric structure of PECPS. The symmetric unit contains one monomer, and in the crystal, the monomer binds another monomer with a crystallographic two-fold axis with root-mean-square deviations of 0.20 Å, forming a biologically active, symmetric dimer. Chains A and B are represented in white and blue, respectively. PECPS adopts the $\alpha\beta\alpha\beta\alpha$-fold architecture in the upper domain. The final model is composed of residues 7-391 of each monomer and contains an independent active site with the catalytic triad, Cys166, His305, and Asn338 highlighted in black, and the Met139 residues protruding into the adjoining monomer are highlighted in magenta. PECPS is structurally related to HsPKS1 from *Huperzia serrata* (PDB entry 3AWJ, Z score = 62.9, rmsd of 0.9 Å over 380 residues aligned with a sequence identity of 56%), based on a homology search using the Dali program.

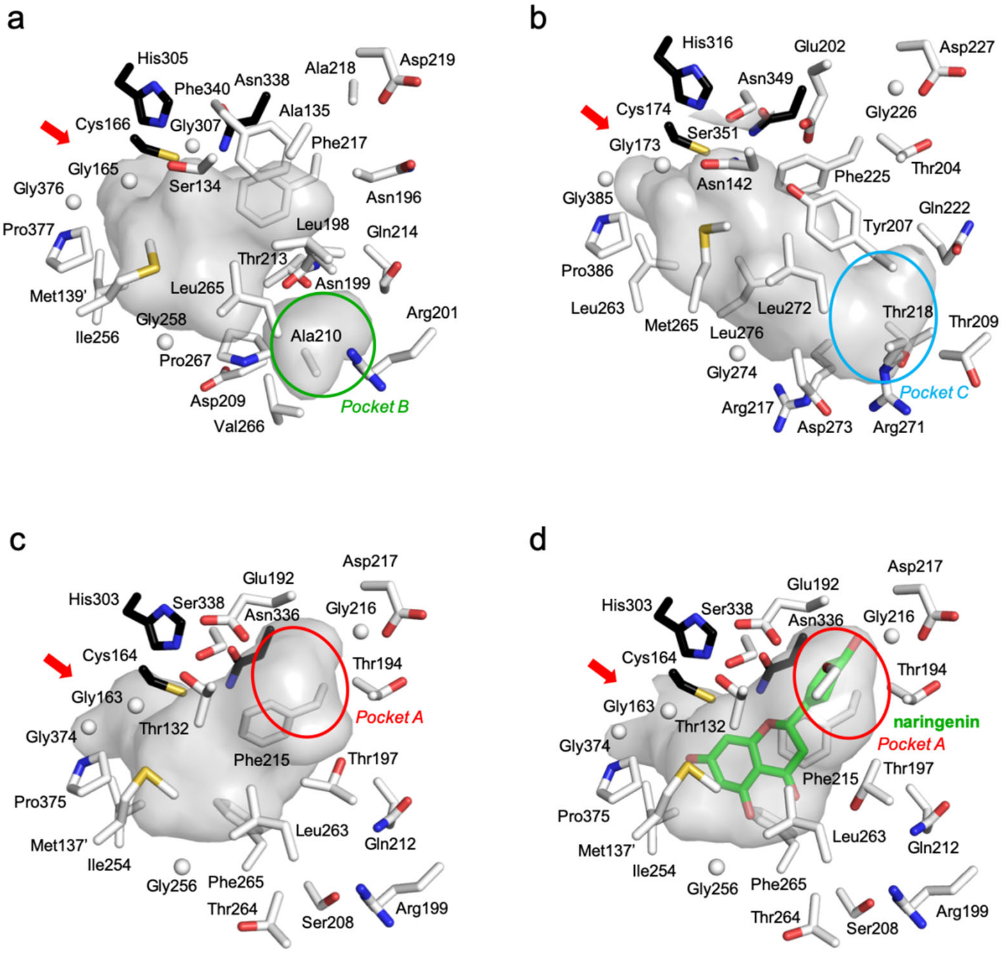

Figure 24. Comparison of the active site topologies in the crystal structure of PECPS wild type, CUS, and MsCHS apo, and MsCHS complexed with naringenin. Surface models represented the wall of the active site for PECPS wild-type (panel **a**, PDB ID: 7C5V), CUS (panel **b**, PDB ID: 3ALE), and MsCHS apo (panel **c**, PDB ID: 1CGK), and MsCHS complexed with naringenin (panel **d**, PDB ID: 1BI5), respectively. The Cys-His-Asn catalytic triad and the naringenin are shown as black and green stick models.

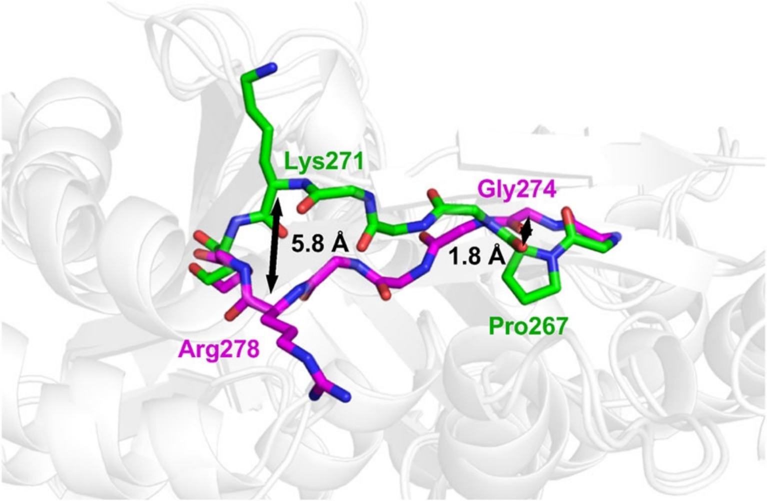

Figure 25. Comparison of the backbones of PECPS and CUS. Residues Val266 to Ile273 of PECPS (green) are compared to residues Asp273 to Val280 of CUS (magenta). The Cα atom of PECPS Lys271 is located at a 5.8 Å distance from that of CUS Arg278. The Cα atom of PECPS Pro267 is located at a 1.8 Å distance from that of CUS Gly274.

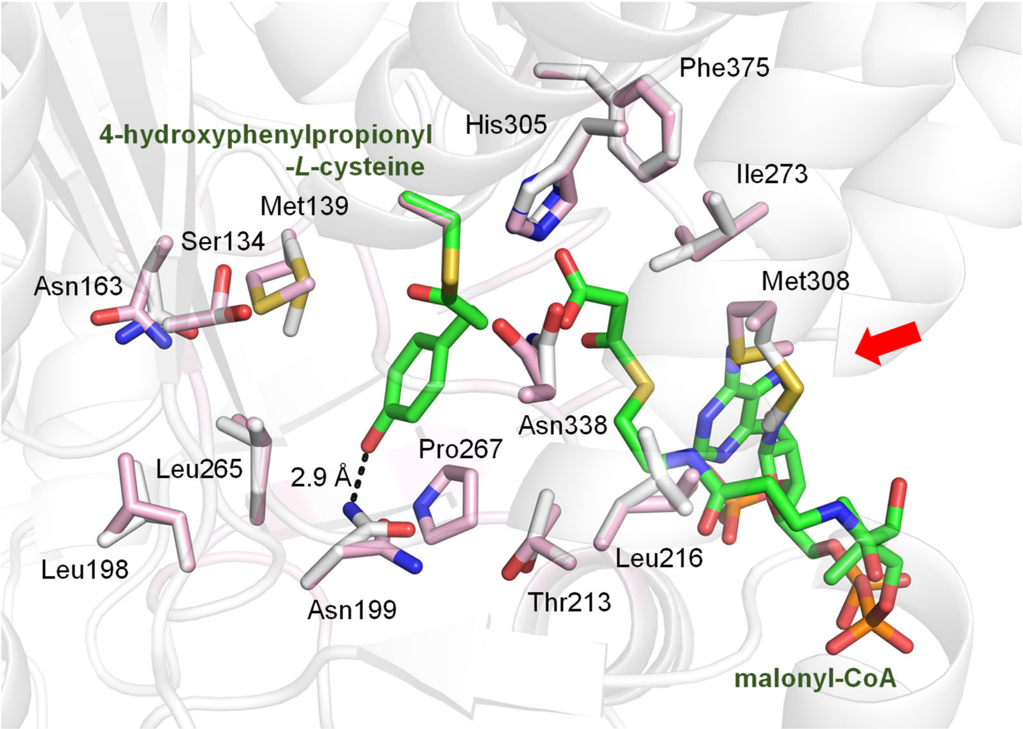

Figure 26. Superpose image of PECPS docking model complexed with 4-hydroxyphenylpropionyl-*L*-cysteine and malonyl-CoA. Superimposition of the PECPS wild type apo structure (PDB ID:7FFA) with the docking model of PECPS complexed with 4-hydroxyphenylpropionyl-*L*-cysteine and malonyl-CoA revealed the slight shift of Asn199, which was at 2.9 Å distance from the 4-hydroxyphenylpropionyl-*L*-cysteine in the model structure. The PECPS wild-type apo structure and docking model of PECPS wild-type complexed with 4-hydroxyphenylpropionyl-*L*-cysteine are shown as white and light pink stick models, respectively. 4-Hydroxyphenylpropionyl-*L*-cystein and malonyl-CoA are shown as green stick models. The entrance to the catalytic cavity is indicated with a red arrow.

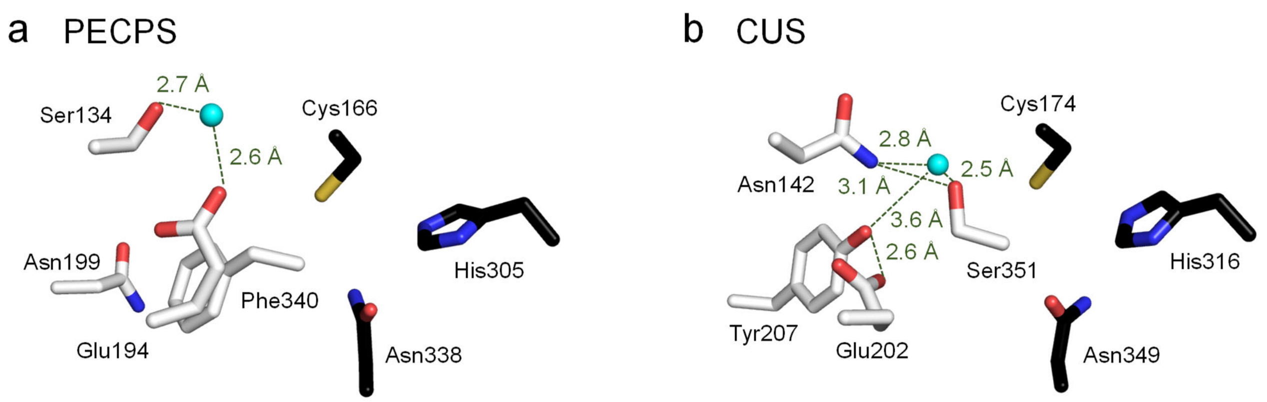

Figure 27. Close-up views of the electronic hydrogen bond network of PECPS (panel a, PDB ID: 7FFA) and CUS (panel b, PDB ID: 3ALE). The water molecules and the hydrogen bonds are indicated with light blue spheres and green dotted lines.

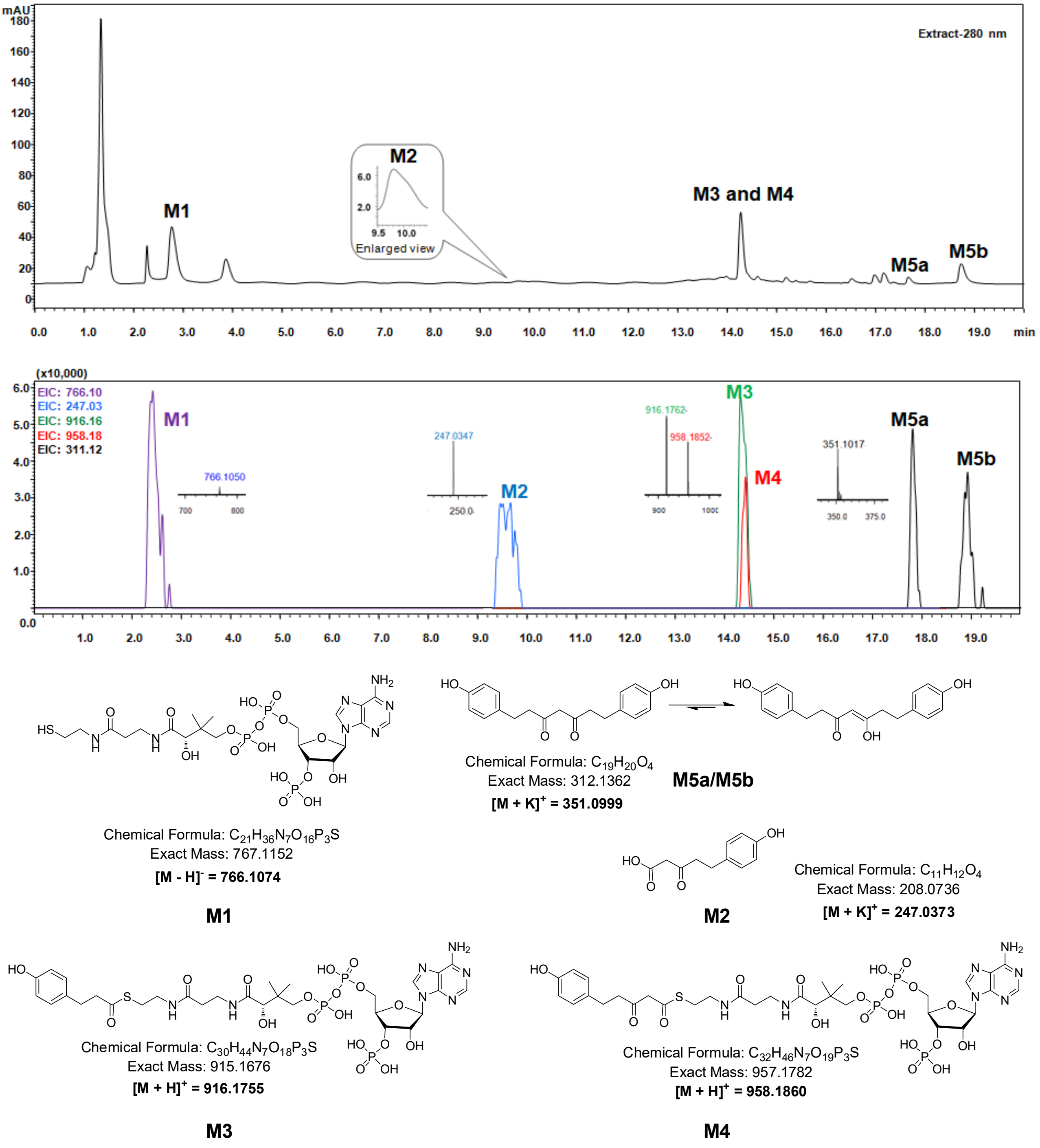

Figure 28. HPLC chromatogram (280 nm) and HRESI MS data of 4-hydroxyphenylpropionyl-β-diketide-CoA, produced by PECPS from the condensation of 4-hydroxyphenylpropionyl-CoA and one molecule of malonyl-CoA. Although the product 4-hydroxyphenylpropionyl-β-diketide-CoA and the substrate 4-hydroxyphenylpropionyl-CoA could not be separated, the presence of the $[M + H]^+$ ion peak at *m/z* 958.1852 suggested the formation of 4-hydroxyphenylpropionyl-β-diketide-CoA (**M4**). In contrast, the presence of the $[M + H]^+$ ion peak at *m/z* 916.1762 was assigned to the substrate, 4-hydroxyphenylpropionyl-CoA (**M3**). In addition, free coenzyme A (**M1**), 4-hydroxyphenylpropionyl-β-diketide acid (**M2**), and the final product tetrahydrobisdemethoxycurcumin (**M5a**/**M5b,** keto-, enol-form) could also be detected in the reaction solution.

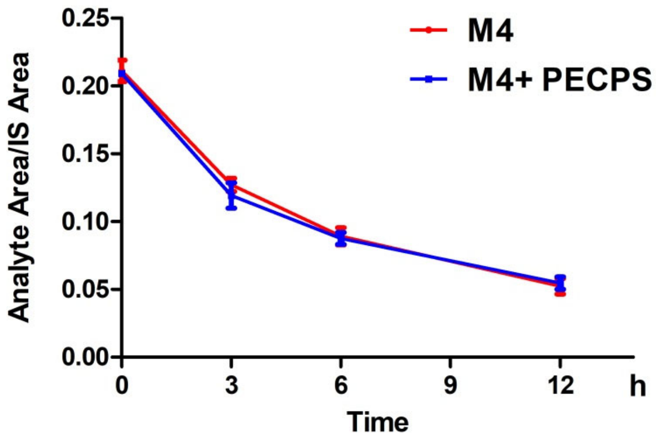

Figure 29. The time course of the hydrolysis of 4-hydroxyphenylpropionyl-*β*-diketide-CoA (M4) in KPB buffer (pH 7.5) at 37 °C with and without PECPS. The diketide-CoA intermediate **M4** could be spontaneously hydrolyzed in buffer (pH 7.5) at 37 °C, and about 75% of **M4** was hydrolyzed after 12 hours, while the hydrolysis rate of **M4** with PECPS was the same as that without PECPS, suggesting the non-enzymatically hydrolysis of **M4** in the reaction buffer. Data represent the mean ± SD (n = 3).

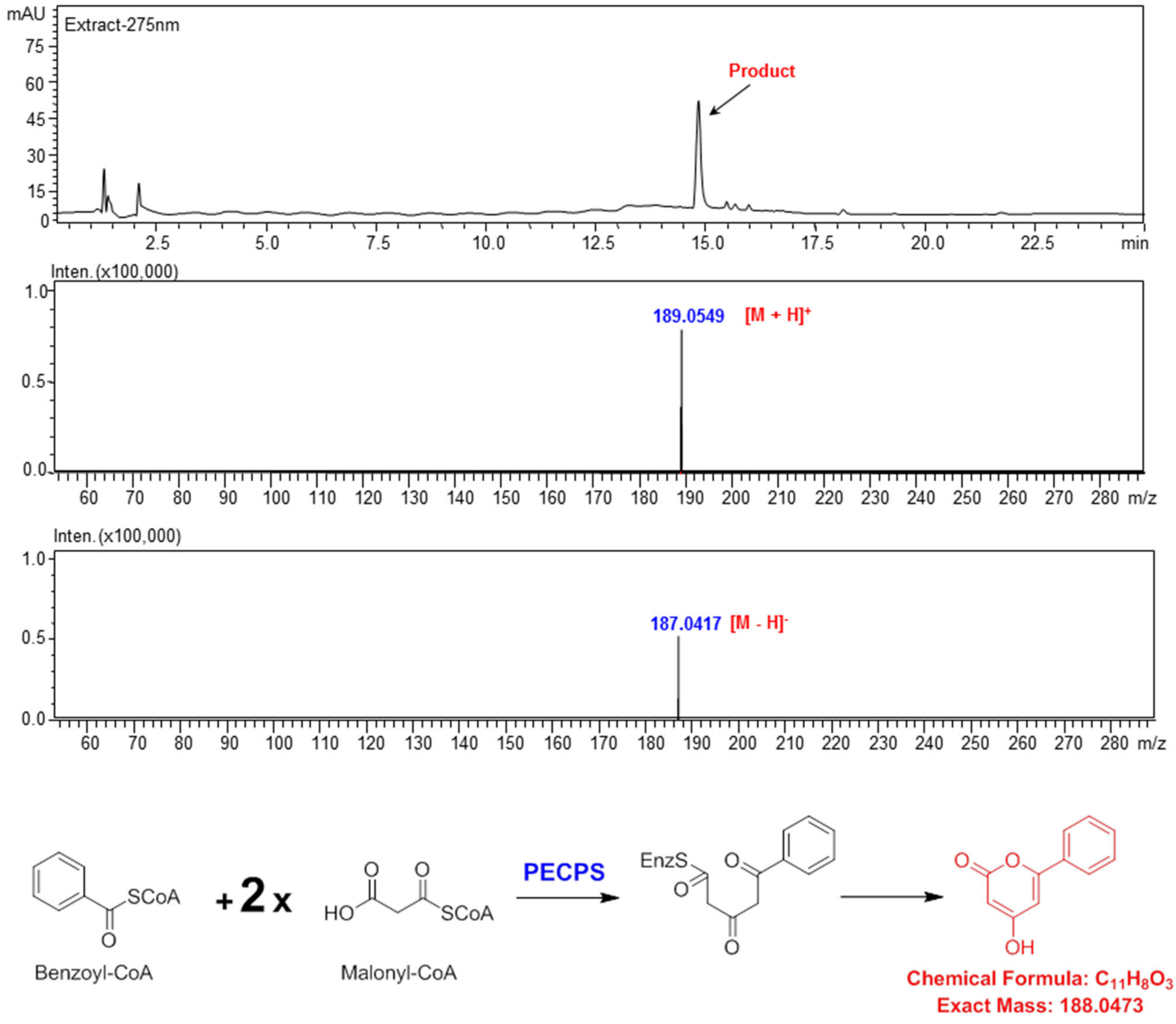

Figure 30. HPLC chromatogram (UV, 275 nm) and MS data of benzoyltriketide pyrone produced by PECPS from the condensation of benzoyl-CoA and two molecules of malonyl-CoA. Benzoyl-β-diketide-CoA and benzoyl-β-diketide acid could not be detected from the reaction mixture (incubated for 5 min, 15 min, 1 h, 3 h, and 5 h, respectively) by LC-MS. However, benzoyltriketide pyrone could be extracted by ethyl acetate from the reaction mixture (incubated for 5h). The HPLC chromatogram (UV, 275 nm) was recorded on a Shim-pack XR-ODS II (100 mm × 2.0 mm, I.D., 5 μm) eluted with 5 mM ammonium formate and acetonitrile in a gradient program: 0–10 min, 5% acetonitrile; 10–25 min, 5%–100% acetonitrile; flow rate: 0.2 mL/min.

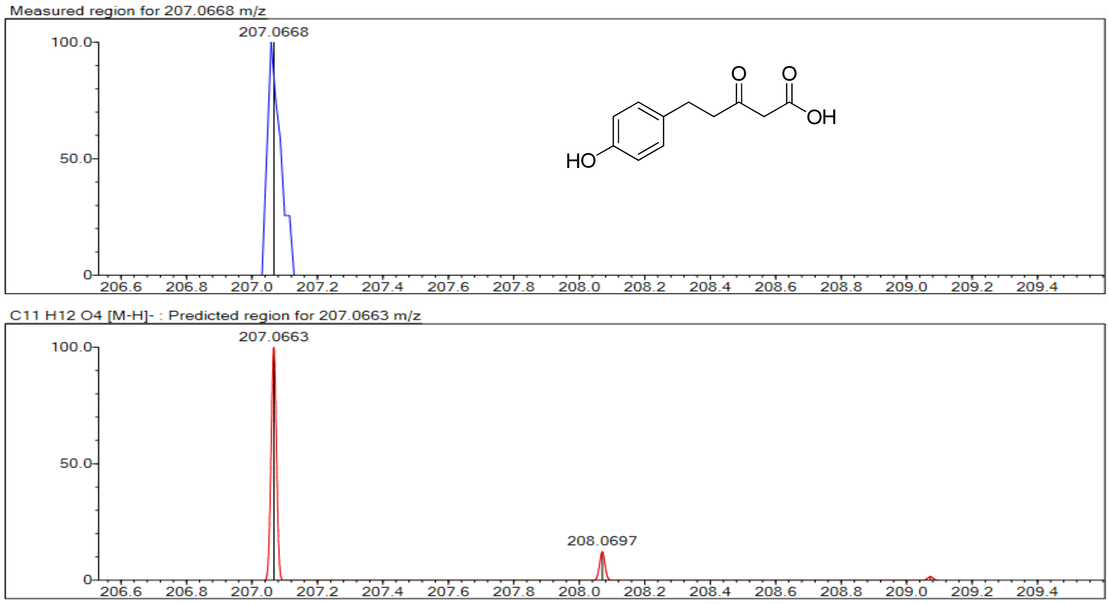

Rank	Score	Formula (M)	Ion	Meas. m/z	Pred. m/z	Df. (mDa)	Df. (ppm)	Iso	DBE
2	0.00	C11 H12 O4	[M-H]-	207.0668	207.0663	0.5	2.41	0.00	6.0

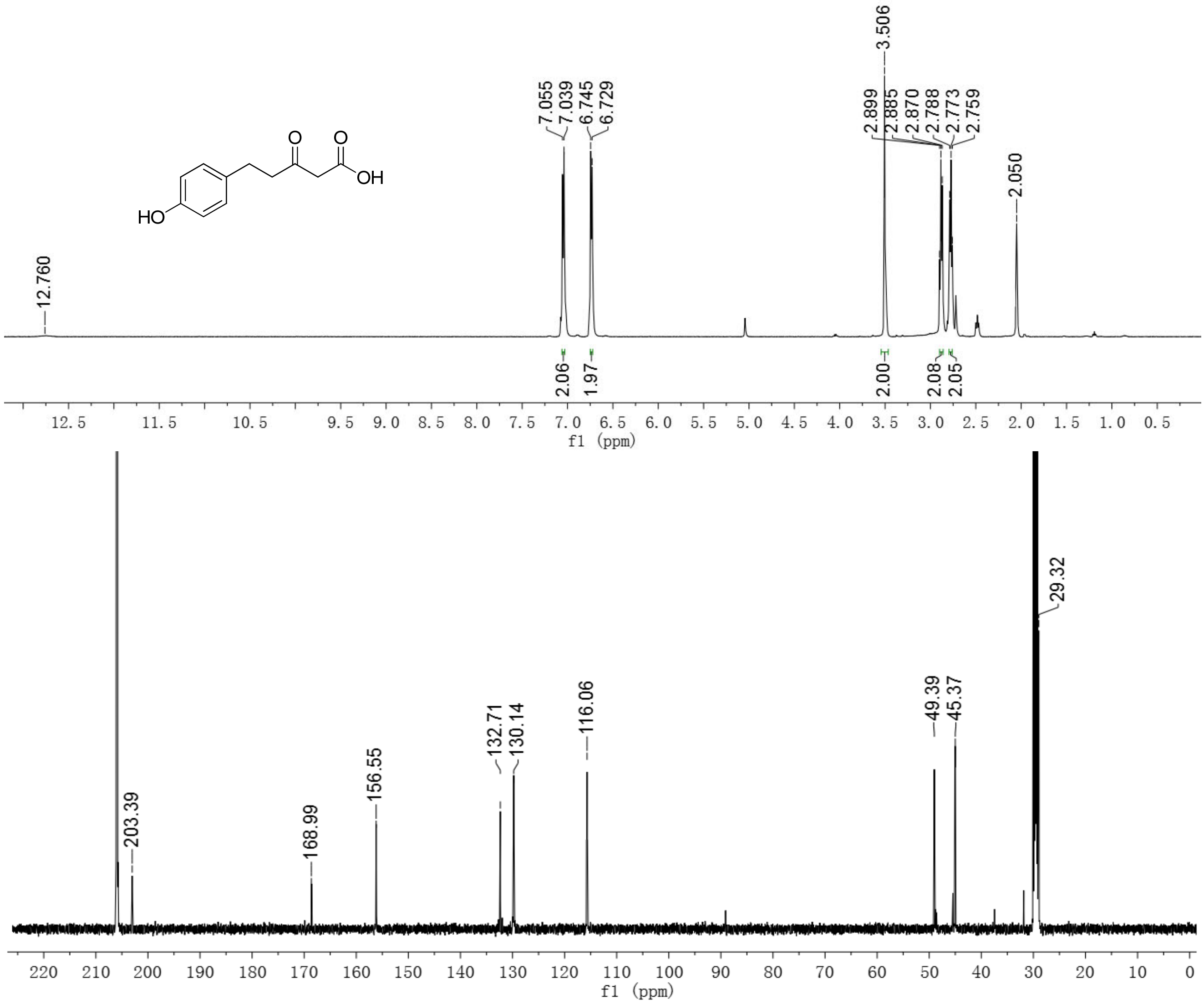

Figure 31. The HRESI MS, ^{1}H NMR, and ^{13}C NMR spectra of 4-hydroxyphenylpropionyl-*β*-diketide acid in acetone-*d*$_6$.

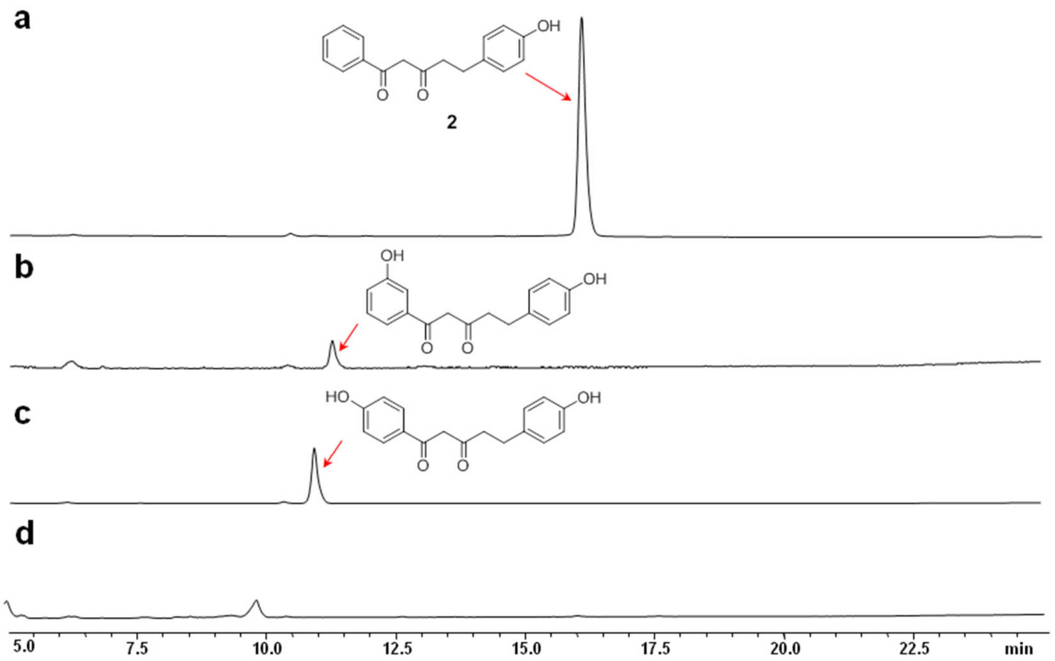

Figure 32. PECPS catalyzes the formation of C_6-C_5-C_6 scaffolds. **a** From 4-hydroxyphenylpropionyl-*β*-diketide acid and benzoyl-CoA. **b** From 4-hydroxyphenylpropionyl-*β*-diketide acid and 3-hydroxybenzoyl-CoA. **c** From 4-hydroxyphenylpropionyl-*β*-diketide acid and 4-hydroxybenzoyl-CoA. **d** From 4-hydroxyphenylpropionyl-*β*-diketide acid and 2-hydroxybenzoyl-CoA. HPLC chromatograms were recorded at 290 nm, and the enzymatic products were assigned by their HRESI MS data. The C_6-C_5-C_6 scaffold could not be produced by PECPS from 2-hydroxybenzoyl-CoA (reaction d), and the small peak at about 10 min in panel D was also present in the reaction with the boiled enzyme.

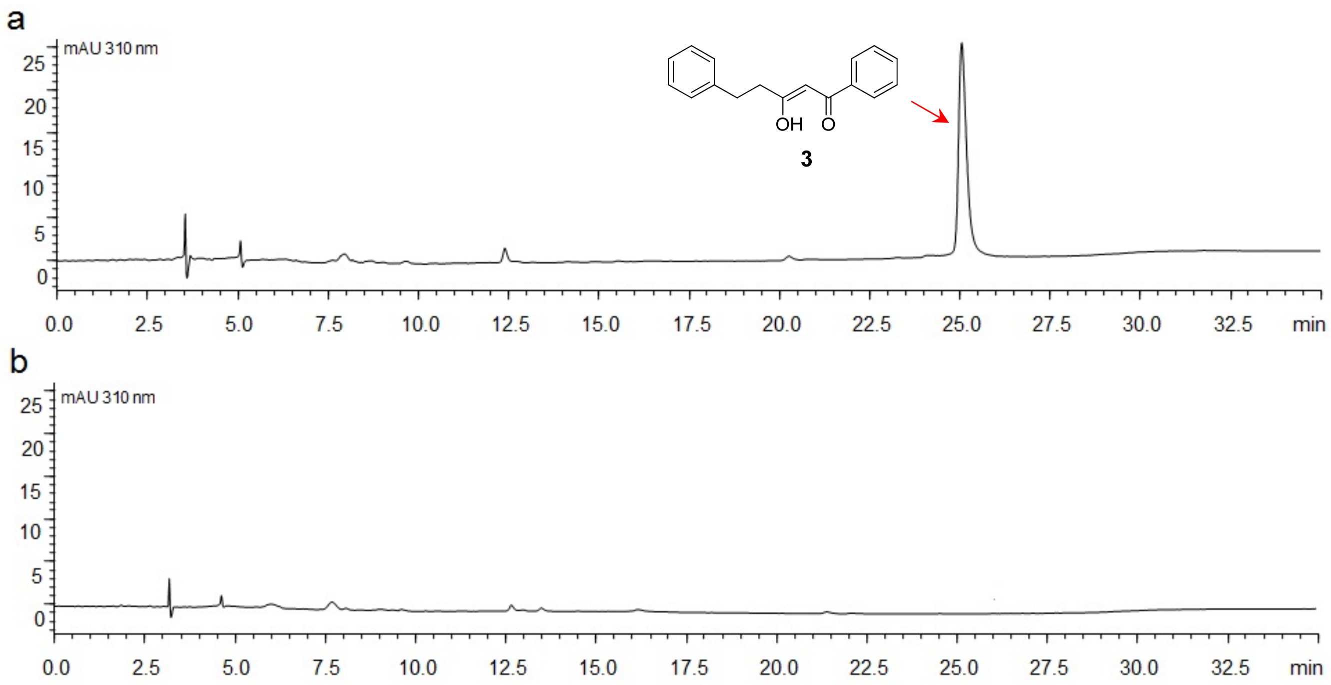

Figure 33. HPLC chromatogram for the formation of the C_6-C_5-C_6 scaffold of 1,5-diphenylpentane-1,3-dione (3) from benzoyl-*β*-diketide acid and phenylpropionyl-CoA. a PECPS; **b** Boiled PECPS.

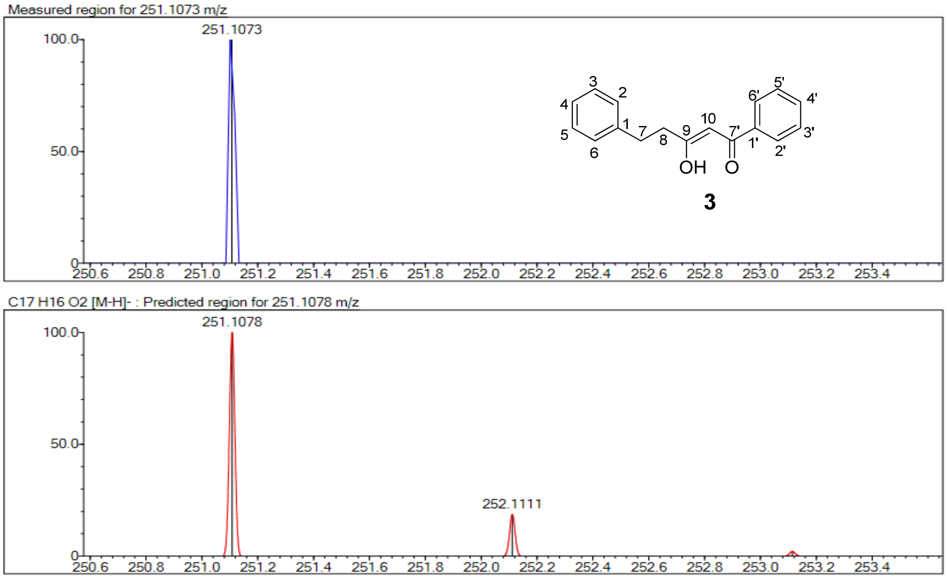

Rank	Score	Formula (M)	Ion	Meas. m/z	Pred. m/z	Df. (mDa)	Df. (ppm)	Iso	DBE
1	0.00	C17 H16 O2	[M-H]-	251.1073	251.1078	-0.5	-1.99	0.00	10.0

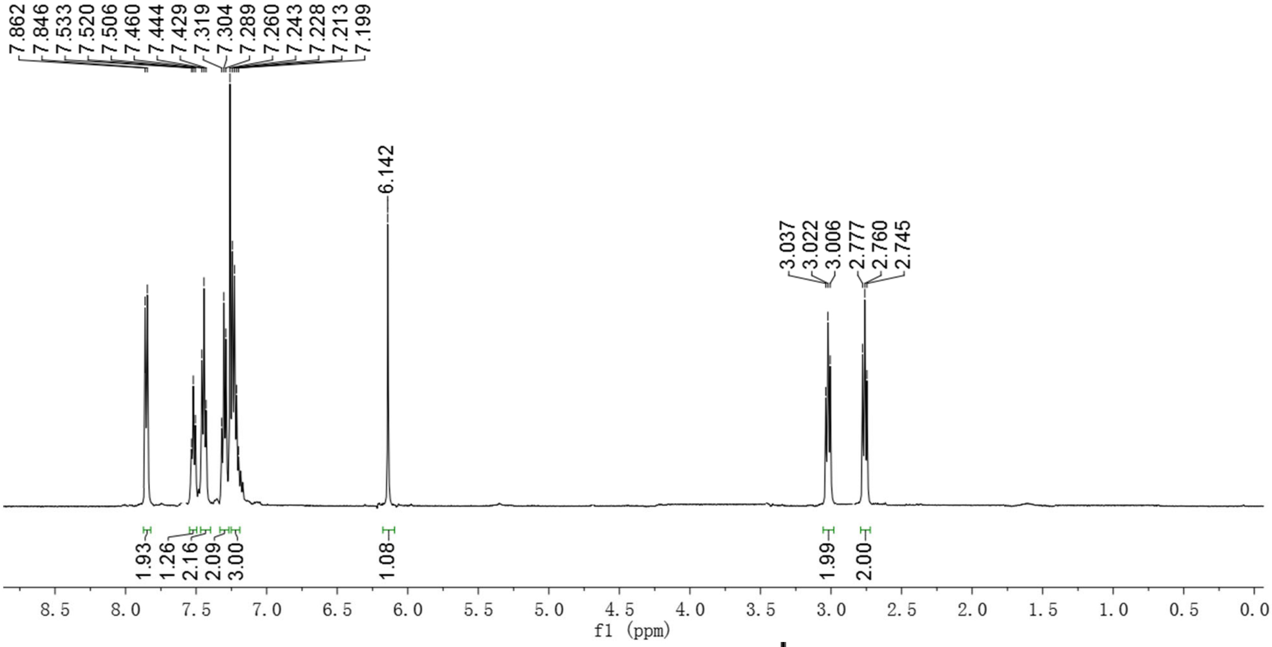

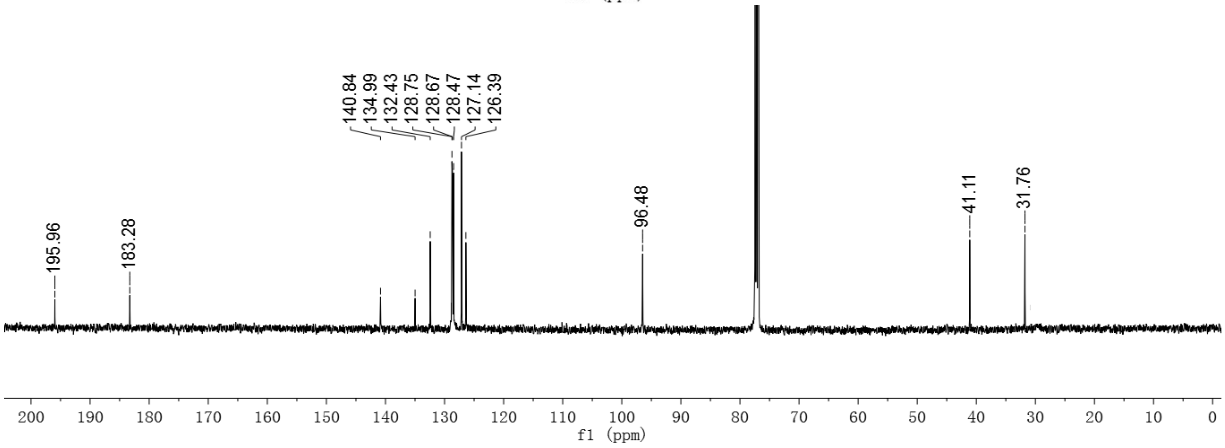

Figure 34. The HRESI MS, ^{1}H NMR, and ^{13}C NMR spectra of 3 in $CDCl_3$

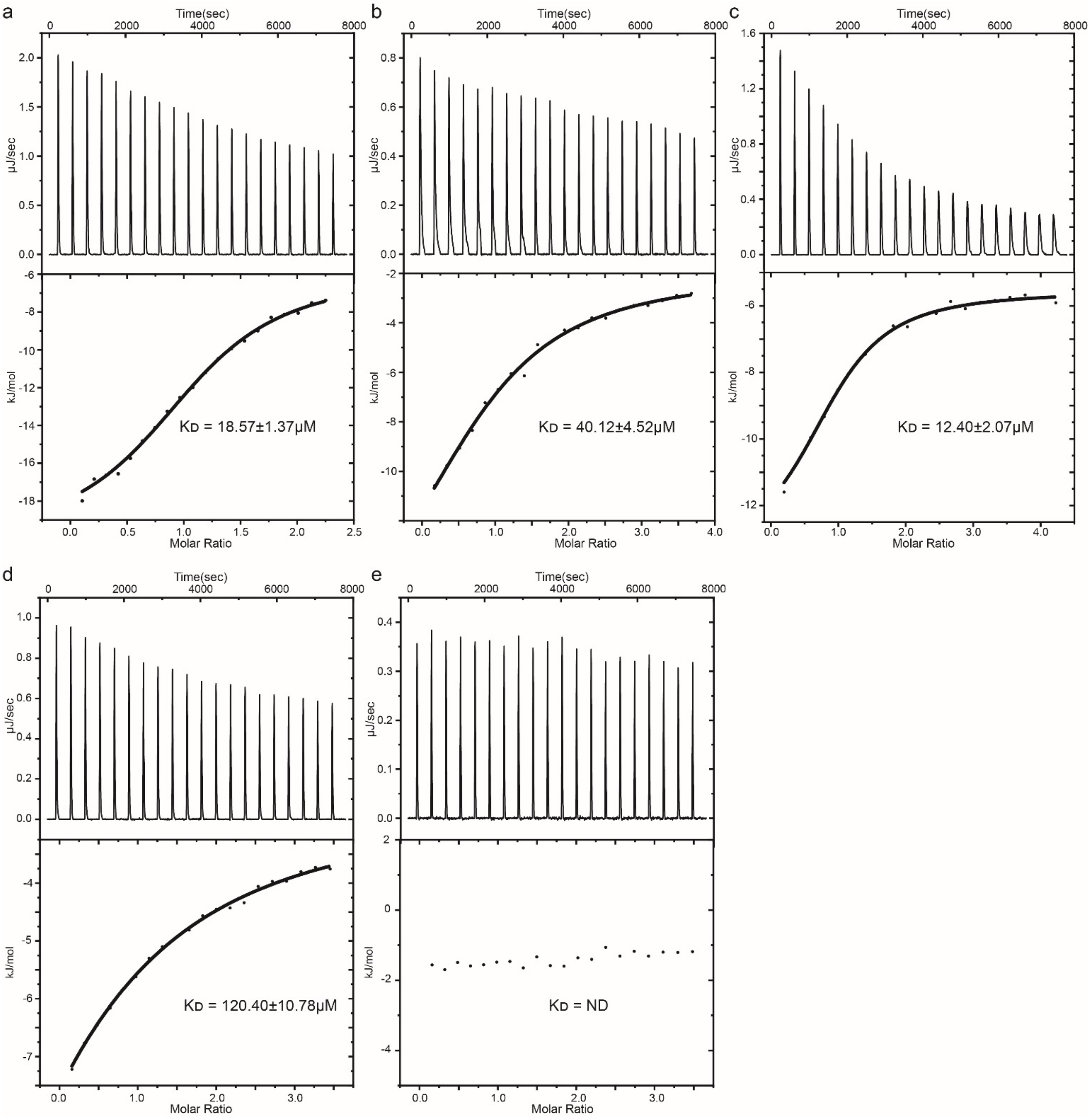

Figure 35. Representative ITC thermograms and isotherm plots for the interaction of 4-hydroxyphenylpropionyl -CoA and different proteins. a wild PECPS; **b** PECPS A210E; **c** PECPS F340W; **d** PECPS N199L; **e** PECPS N199F. Each experiment was independently repeated three times. Fitting curve for PECPS N199F is not presented due to its very weak binding ability with 4-hydroxyphenylpropionyl-CoA (K_D value is close to 1000 μM). Data represent the mean ± SD (n = 3).

Supplementary Tables

Supplementary Table 1. NMR data of compounds 1–3

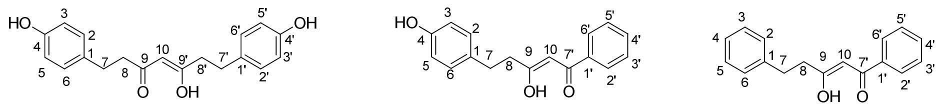

1 **2** **3**

No.	1^a		2^b		3^a	
	δ_H	δ_C	δ_H	δ_C	δ_H	δ_C
1		133.2		132.7		140.8
2,6	7.04 (d, J = 8.0 Hz)	129.6	7.07 (d, J = 8.5 Hz)	130.1	7.24 (m)	127.1
3,5	6.75 (d, J = 8.0 Hz)	115.5	6.71 (d, J = 8.5 Hz)	116.0	7.30 (t, J = 8.0 Hz)	128.7
4		154.1		156.6	7.21 (m)	126.4
7	2.84 (t, J = 7.5 Hz)	30.8	2.89 (t, J = 7.5 Hz)	31.8	3.02 (t, J = 7.5 Hz)	31.8
8	2.53 (t, J = 7.5 Hz)	40.4	2.71 (t, J = 7.5 Hz)	42.1	2.76 (t, J = 7.5 Hz)	41.1
9		193.2		197.4		196.0
10	5.38 (s)	100.0	6.29 (s)	97.2	6.14 (s)	96.5
1'		133.2		135.9		135.0
2', 6'	7.04 (d, J = 8.0 Hz)	129.6	7.46 (br.d, J = 8.0 Hz)	127.8	7.86 (br.d, J = 8.0 Hz)	128.5
3', 5'	6.75 (d, J = 8.0 Hz)	115.5	7.90 (t, J = 8.0 Hz)	129.5	7.44 (t, J = 8.0 Hz)	128.8
4'		154.1	7.54 (m)	133.2	7.52 (m)	132.4
7'	2.53 (t, J = 7.5 Hz)	30.8		183.8		183.3
8'	2.84 (t, J = 7.5 Hz)	40.4				
9'		193.2				

[a]Data were measured in $CDCl_3$ (500 MHz for ^1H, 125 MHz for ^{13}C);
[b]Data were measured in CD_3OD (500 MHz for ^1H, 125 MHz for ^{13}C).

Supplementary Table 2. ^{1}H NMR data of compounds C01–C06

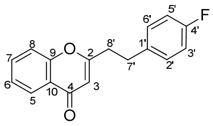

C01

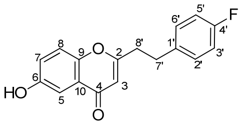

C02

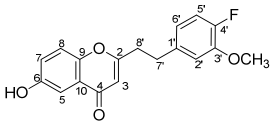

C03

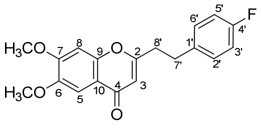

C04

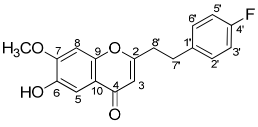

C05

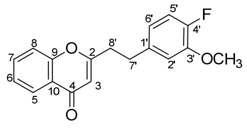

C06

No.	C01[a]	C02[a]	C03[b]	C04[c]	C05[c]	C06[c]
3	6.18 (s)	6.10 (s)	6.13 (s)	6.12 (s)	6.38 (s)	6.15 (s)
5	8.10 (br.d, *J* = 8.0 Hz)	7.37 (d, *J* = 3.0 Hz)	7.26 (d, *J* = 2.5 Hz)	7.50 (s)	7.64 (s)	8.17 (br.dd, *J* = 8.0, 1.5 Hz)
6	7.48 (br.t, *J* = 8.0 Hz)					6.98 (m)
7	7.79 (br.t, *J* = 8.5 Hz)	7.21~7.25 (overlapped)	7.20 (dd, *J* = 8.0, 2.5 Hz)			7.66 (m)
8	7.57 (br.d, *J* = 8.5Hz)	7.43 (d, *J* = 8.0 Hz)	7.47 (d, *J* = 8.0 Hz)	6.85 (s)	6.89 (s)	6.77 (br.dd, *J* = 8.5, 2.0 Hz)
2'	7.25 (m)	7.21~7.25 (overlapped)	7.05~7.10 (overlapped)	7.15 (m)	7.14 (m)	7.30~7.45 (overlapped)
3'	7.00 (t, *J* = 8.0 Hz)	6.98 (t, *J* = 8.0 Hz)		6.97 (t, *J* = 8.5 Hz)	6.98 (t, *J* = 8.5 Hz)	
5'	7.00 (t, *J* = 8.0 Hz)	6.98 (t, *J* = 8.0 Hz)	7.05~7.10 (overlapped)	6.97 (t, *J* = 8.5 Hz)	6.98 (t, *J* = 8.5 Hz)	7.30~7.45 (overlapped)
6'	7.25 (m)	7.21~7.25 (overlapped)	6.76~6.81 (m)	7.15 (m)	7.14 (m)	6.70~6.73 (m)
7'	3.10 (t, *J* = 7.0 Hz)	3.07 (t, *J* = 7.5 Hz)	2.90~ 3.00 (overlapped)	3.02 (t, *J* = 7.5 Hz)	3.06 (t, *J* = 7.0 Hz)	3.02 (t, *J* = 7.0 Hz)
8'	3.03 (t, *J* = 7.0 Hz)	2.98 (t, *J* = 7.5 Hz)	2.90~ 3.00 (overlapped)	2.88 (t, *J* = 7.5 Hz)	2.97 (t, *J* = 7.0 Hz)	2.91 (t, *J* = 7.0 Hz)
6-OCH_3				3.96 (s)		
7-OCH_3				3.98 (s)	4.03 (s)	
3'-OCH_3			3.76 (s)			3.82 (s)

[a]Data were measured in CD_3OD, 500 MHz;
[b]Data were measured in DMSO-d_6, 500 MHz;
[c]Data were measured in $CDCl_3$, 500 MHz.

Supplementary Table 3. ^{13}**C NMR data of compounds C01–C06**

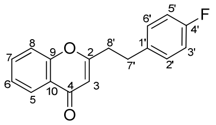

C01

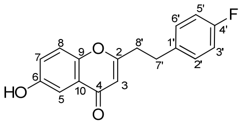

C02

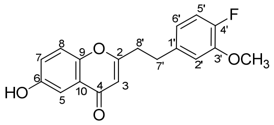

C03

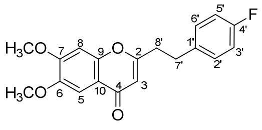

C04

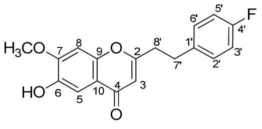

C05

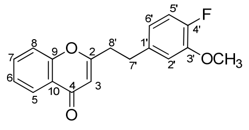

C06

No.	C01[a]	C02[a]	C03[b]	C04[c]	C05[c]	C06[c]
2	171.5	171.0	169.5	167.5	168.7	168.4
3	110.8	109.8	109.9	109.8	109.0	110.5
4	180.5	180.5	177.9	177.6	177.6	178.5
5	126.6	108.7	108.7	104.5	108.1	125.9
6	126.2	152.0	150.8	147.6	144.6	125.3
7	135.5	124.5	124.0	152.6	152.8	133.9
8	119.2	120.4	120.6	99.6	99.1	118.0
9	158.1	156.4	155.9	154.5	152.5	156.6
10	124.3	125.1	125.2	117.0	117.0	123.9
1'	137.2	137.3	138.1	135.5	135.4	136.2
2'	131.2, 131.1	131.2, 131.1	115.2	129.9, 129.8	129.9, 129.8	113.7
3'	116.2, 116.1	116.2, 116.0	148.1, 148.0	115.7, 115.5	115.7, 115.6	147.8,147.7
4'	164.0, 162.1	164.0, 162.1	152.2, 150.6	162.7, 160.7	162.7, 160.8	152.4, 150.5
5'	116.2, 116.1	116.2, 116.0	116.8, 116.7	115.7, 115.5	115.7, 115.6	116.3, 116.2
6'	131.2, 131.1	131.2, 131.1	121.6, 121.5	129.9, 129.8	129.9, 129.8	120.6, 120.5
7'	33.1	33.2	33.0	32.4	32.5	32.9
8'	37.1	37.1	36.1	36.3	36.4	36.4
6-OCH_3				56.5		
7-OCH_3				56.6	56.7	
3'-OCH_3			57.0			56.4

[a]Data were measured in CD_3OD, 125 MHz;
[b]Data were measured in DMSO-d_6, 125 MHz;
[c]Data were measured in $CDCl_3$, 125 MHz.

Supplementary Table 4. ITC-measured thermodynamic characteristics of the interactions between PECPS mutants and 4-hydorxyphenylpropionyl-CoA.

	PECPS	PECPS A210E	PECPS F340W	PECPS N199L	PECPS N199F
K_D (μM)	18.57 ± 1.37	40.12 ± 4.52	12.40 ± 2.07	120.40 ± 10.78	ND*
n	1.10 ± 0.12	0.99 ± 0.02	0.91 ± 0.06	0.87 ± 0.06	ND*
ΔH (KJ/mol)	-12.42 ± 1.78	-15.73 ± 1.35	-7.19 ± 0.74	-19.70 ± 1.68	ND*
Ka (mM^{-1})	50.06 ± 4.14	25.12 ± 2.98	82.07 ± 12.96	8.35 ± 0.71	ND*
-TΔS (KJ/mol)	-15.68 ± 1.94	-10.39 ± 1.66	-21.97 ± 1.17	3.57 ± 1.51	ND*
ΔG (KJ/mol)	-20.10 ± 0.20	-26.12 ± 0.30	-29.16 ± 0.42	-23.28 ± 0.23	ND*
ΔS (J/mol·K)	50.56 ± 6.28	33.49 ± 5.34	70.83 ± 3.75	11.50 ± 4.86	ND*

Each experiment was independently repeated three times. Data represent the mean ± SD (n = 3). The representative ITC thermograms and isotherm plots could be found in supplementary **Figure 35**. *ND means the mutant N199F indicated very weak binding ability with the substrate (K_D value is close to 1000 μM), which is challenging to get its accurate K_D value by fitting a reasonable curve.

Supplementary Table 5. Primers used for Site-directed mutagenesis

**Mutants**	**Primer sequences**
A210E	5'-TCGGAGACCCACATCGACGAGCTCATAACTCAATCT-3'
	5'-AGATTGAGTTATGA GCTCGTCGATGTGGGTCTCCGA-3'
F340W	5'-GTGAGTTTGGCAATATGTGGAGTGCCACCG-3'
	5'-CCACATATTGCCAAACTCACTAAGCACGTG-3'
N199L	5'-CGGAGGCCAACCTACTCCTCTTCCGGGGCCCG-3'
	5'-GAGGAGTAGGTTGGCCTCCGAGCAGACGACAAG-3'
N199F	5'-CGGAGGCCAACCTACTCTTCTTCCGGGGCCCG-3'
	5'-GAAGAGTAGGTTGGCCTCCGAGCAGACGACAAG-3'

Supplementary Table 6. Crystal data collection, phasing, and refinement statistics

Data collection	**PECPS**	**PECPS A210E**	**PECPS F340W**	**PECPS N199L**	**PECPS N199F**
Space group	*C*2	$P2_1$	*C*2	*C*2	*C*2
a, b, c (Å)	147.0, 65.3, 108.0	108.0, 126.9, 118.2	147.2, 65.1, 108.6	146.4, 65.2, 108.4	146.6, 65.1, 108.0
α, β, γ (°)	β = 127.8	β = 99.4	β = 128.2	β = 128.1	β = 128.1
Resolution (Å)	42.65-1.98 (2.03-1.98)	49.23-2.61 (2.65-2.61)	42.66-2.40 (2.49-2.40)	42.64-2.20 (2.27-2.20)	42.49-2.30 (2.38-2.30)
R_{merge} (%)	4.8 (61.2)	14.1 (74.8)	8.5 (49.4)	4.3 (36.0)	5.8 (52.0)
$<I / (\sigma I)>$	21.9 (2.8)	6.0 (1.6)	8.2 (2.2)	15.1 (3.3)	17.7 (3.4)
CC/2	0.999 (0.869)	0.991 (0.759)	0.996 (0.881)	0.999 (0.954)	0.999 (0.937)
Completeness (%)	99.9 (98.7)	99.3 (87.7)	99.7 (100.0)	99.7 (99.7)	99.9 (100.0)
Redundancy	6.9 (5.8)	3.9 (3.7)	3.6 (4.0)	4.4 (4.5)	6.9 (7.2)
Refinement					
Resolution (Å)	42.65-1.98	49.13-2.61	42.66-2.40	40.51-2.20	41.70-2.30
No. Reflections	54071	94842	31634	40833	35782
R_{work}/R_{free} (%)	19.2/23.7	22.0/26.9	20.7/25.8	21.3/25.8	18.9/22.1
No. Atoms					
Proteins	5904	23913	5908	5878	5894
Ligand/ion	-	90	-	-	-
Water	293	404	121	88	129
B-factor	37.0	43.0	48.2	61.8	58.9
Root mean square deviations					
Bond lengths (Å)	0.007	0.002	0.003	0.008	0.004
Bond Angles (°)	0.855	0.497	0.538	0.916	0.745
Ramachandran plot (%)					
Favored/Allowed/Outlier	96.3/3.7/0.0	96.1/3.9/0.0	95.0/5.0/0.0	95.4/4.4/0.1	95.2/4.8/0.0
PDB Accession Code	7FFA	7FFC	7FFI	7FFH	7FFG

One crystal was used for data collection. Values in parentheses are for highest-resolution shell.

Supplementary Table 7. Results of docking to active centers of wild type PECPS for different ligands

Entry	Residue 166	Ligand	Free binding energy (kcal/mol)
1	4-hydroxyphenylpropionyl-*L*-cysteine	malonyl-CoA	-6.8
2	*L*-cysteine	4-hydroxyphenylpropionyl-β-diketide-CoA	-7.9
3	benzoyl-*L*-cysteine	phenylpropionyl-β-diketide acid	-7.6
4	*L*-cysteine	C_6-C_5-C_6 scaffold product	-5.9
