# Supplementary material for: Identification of a diarylpentanoid-producing polyketide synthase revealing an unusual biosynthetic pathway of 2-(2-phenylethyl)chromones in agarwood
Source: Nat Commun. 2022 Jan 17;13:348. doi: 10.1038/s41467-022-27971-z (PMC8764113; doi:10.1038/s41467-022-27971-z)
Supplement: Supplementary file 3 — Reporting Summary [file 41467_2022_27971_MOESM3_ESM.pdf]

nature portfolio

Corresponding author(s): Shepo shi

Last updated by author(s): Dec. 14, 2021

Reporting Summary

Nature Portfolio wishes to improve the reproducibility of the work that we publish. This form provides structure for consistency and transparency in reporting. For further information on Nature Portfolio policies, see our Editorial Policies and the Editorial Policy Checklist.

Statistics

For all statistical analyses, confirm that the following items are present in the figure legend, table legend, main text, or Methods section.

n/a	Confirmed	
☐	☒	The exact sample size (*n*) for each experimental group/condition, given as a discrete number and unit of measurement
☐	☒	A statement on whether measurements were taken from distinct samples or whether the same sample was measured repeatedly
☐	☒	The statistical test(s) used AND whether they are one- or two-sided<br>*Only common tests should be described solely by name; describe more complex techniques in the Methods section.*
☐	☒	A description of all covariates tested
☐	☒	A description of any assumptions or corrections, such as tests of normality and adjustment for multiple comparisons
☐	☒	A full description of the statistical parameters including central tendency (e.g. means) or other basic estimates (e.g. regression coefficient) AND variation (e.g. standard deviation) or associated estimates of uncertainty (e.g. confidence intervals)
☐	☒	For null hypothesis testing, the test statistic (e.g. *F*, *t*, *r*) with confidence intervals, effect sizes, degrees of freedom and *P* value noted<br>*Give P values as exact values whenever suitable.*
☒	☐	For Bayesian analysis, information on the choice of priors and Markov chain Monte Carlo settings
☒	☐	For hierarchical and complex designs, identification of the appropriate level for tests and full reporting of outcomes
☒	☐	Estimates of effect sizes (e.g. Cohen's *d*, Pearson's *r*), indicating how they were calculated

Our web collection on statistics for biologists contains articles on many of the points above.

Software and code

Policy information about availability of computer code

Data collection	qRT-PCR: CFX Manager Software for Bio-Rad CFX Real -Time PCR System version 3.0)
Data analysis	Western blot: Fuji distribution of Image J (version 2.0.0) and Prism 8 (GraphPad Sofware Inc, version 8.4.3)<br>qRT-PCR: Prism 8 (GraphPad Software Inc, version 8.4.3)<br>Mass spectrometric qualitative, Mass spectrometric quantification, ITC: Origin(OriginLab corp., version 2018)

For manuscripts utilizing custom algorithms or software that are central to the research but not yet described in published literature, software must be made available to editors and reviewers. We strongly encourage code deposition in a community repository (e.g. GitHub). See the Nature Portfolio guidelines for submitting code & software for further information.

Data

Policy information about availability of data

All manuscripts must include a data availability statement. This statement should provide the following information, where applicable:

- Accession codes, unique identifiers, or web links for publicly available datasets
- A description of any restrictions on data availability
- For clinical datasets or third party data, please ensure that the statement adheres to our policy

The structures of wild PECPS and PECPS A210E, F340W, N199L and N199F have been deposited in the Protein Data Bank under codes 7FFA, 7FFC, 7FFI, 7FFH, and 7FFG , respectively. The GenBank accession number for the nucleotide sequence of PECPS is MH885494. RNA-Seq data that support the findings of this study have been deposited in the National Center for Biotechnology Information (NCBI) Sequence Read Archive (SRA) with accession number SRA319923. The source data underlying Figs. 1, 3a, 3c, 3d, 4i, Supplementary Figs. 7, 20, 21, 29, and 35 are provided as a Source Data file.

Field-specific reporting

Please select the one below that is the best fit for your research. If you are not sure, read the appropriate sections before making your selection.

☒ Life sciences ☐ Behavioural & social sciences ☐ Ecological, evolutionary & environmental sciences

For a reference copy of the document with all sections, see nature.com/documents/nr-reporting-summary-flat.pdf

Life sciences study design

All studies must disclose on these points even when the disclosure is negative.

Sample size	qRT-PCR analysis were performed in three biological replicates.
Data exclusions	No data were excluded.
Replication	Experiments were independently repeated as indicated in the figure legends and could have been successfully replicated. For qRT-PCR and western blot , three biological replicates were taken to verify the reproducibility of the experimental finding.
Randomization	To analysis the expression level of PECPS, three different salt-treated plate or three pecps knockdown lines were selected randomly for western blot and qRT-PCR analysis in Fig.1, Fig.S7 and Fig.S20.
Blinding	No blinding to group allocation was done during data collection and or analysis.

Reporting for specific materials, systems and methods

We require information from authors about some types of materials, experimental systems and methods used in many studies. Here, indicate whether each material, system or method listed is relevant to your study. If you are not sure if a list item applies to your research, read the appropriate section before selecting a response.

Materials & experimental systems

n/a	Involved in the study
☐	☒ Antibodies
☒	☐ Eukaryotic cell lines
☒	☐ Palaeontology and archaeology
☒	☐ Animals and other organisms
☒	☐ Human research participants
☒	☐ Clinical data
☒	☐ Dual use research of concern

Methods

n/a	Involved in the study
☒	☐ ChIP-seq
☒	☐ Flow cytometry
☒	☐ MRI-based neuroimaging

Antibodies

Antibodies used	PECPS Rabbit Polyclonal Antibody,Prepared by Laboratory Animal Center, Institute of genetics and developmental biology, Chinese Academy of Sciences, 1mg/ml, 1:1000;<br>GAPDH Mouse Monoclonal Antibody , 1mg/ml, Proteintech, Catalog Number: 60004-1-lg, 1:20000
Validation	PECPS Rabbit Polyclonal Antibody: Tested Applications: WB; Species Specificity: Aquilaria sinensis.<br>GAPDH Mouse Monoclonal Antibody: Antibody validations were performed by supplier. Tested Applications:FC,IF,IP,WB,ELISA; Cited Applications: ChIP, CoIP, FC, IF,IHC,IP, WB; Species Specificity: human, mouse,rat,yeast, plant; Cited Species: bovine,Branchiostoma belcheri; Caenorhabditis elegans, canine, chicken, Cynomorium songaricum, Deer, duck,frog; Additional validation has also been given in previous publication with PubMed IDs:32999218,33061618 and 32999280.
